# Impact of mechanical cues on key cell functions and cell-nanoparticle interactions

**DOI:** 10.1186/s11671-024-04052-2

**Published:** 2024-06-22

**Authors:** Petra Elblová, Mariia Lunova, Alexandr Dejneka, Milan Jirsa, Oleg Lunov

**Affiliations:** 1 Department of Optical and Biophysical Systems, Institute of Physics of the Czech Academy of Sciences, 18200 Prague, Czech Republic; 2 Faculty of Mathematics and Physics, Charles University, Ke Karlovu 3, 121 16 Prague 2, Czech Republic; 3 Institute for Clinical & Experimental Medicine (IKEM), 14021 Prague, Czech Republic

**Keywords:** Nanomedicine; Nanoparticles, Mechanotransduction, Mechanical cues, Extracellular matrix

## Abstract

In recent years, it has been recognized that mechanical forces play an important regulative role in living organisms and possess a direct impact on crucial cell functions, ranging from cell growth to maintenance of tissue homeostasis. Advancements in mechanobiology have revealed the profound impact of mechanical signals on diverse cellular responses that are cell type specific. Notably, numerous studies have elucidated the pivotal role of different mechanical cues as regulatory factors influencing various cellular processes, including cell spreading, locomotion, differentiation, and proliferation. Given these insights, it is unsurprising that the responses of cells regulated by physical forces are intricately linked to the modulation of nanoparticle uptake kinetics and processing. This complex interplay underscores the significance of understanding the mechanical microenvironment in shaping cellular behaviors and, consequently, influencing how cells interact with and process nanoparticles. Nevertheless, our knowledge on how localized physical forces affect the internalization and processing of nanoparticles by cells remains rather limited. A significant gap exists in the literature concerning a systematic analysis of how mechanical cues might bias the interactions between nanoparticles and cells. Hence, our aim in this review is to provide a comprehensive and critical analysis of the existing knowledge regarding the influence of mechanical cues on the complicated dynamics of cell-nanoparticle interactions. By addressing this gap, we would like to contribute to a detailed understanding of the role that mechanical forces play in shaping the complex interplay between cells and nanoparticles.

## Introduction

Unique physicochemical features of nanomaterials opened a door for their application in various fields including biomedicine [1]. It is feasible to improve disease diagnosis and treatment specificity using designed nanoparticles [1–3]. For instance nanoparticles represent promising formulations that may ameliorate the stability and solubility of encapsulated drugs, improve transport across membranes and affect circulation times to increase safety and efficacy of nano-based medicines [4, 5]. Furthermore, novel nanomaterials (e.g. DNA nanostructures) offer promising foundation of better and safer nanoformulations for various biomedical applications [6–8]. In fact, nanoformulations contributed greatly to vaccine design and efficiency, simultaneously providing a platform that avoids severe adverse reactions [9]. However, nanomaterials can also possess serious toxic effects resulting into adverse drug reactions [10–14]. Additionally, the clinical translation of nanomaterials into nanomedicines available to patients is recognized to be lower than what is expected [1, 15]. The low rate of nanomedicine’s clinical success is generally attributed to translational gap between animal and human studies [1, 15]. On one hand, this gap originates from poor understanding of key differences in physiology and pathology between animal models utilized in preclinical research and humans [1, 16]. On the other hand, lack of clear understanding of fundamental mechanisms of nanomaterials-cell interactions contributes to the problem of clinical translation of nanomaterials [1, 13]. It is worth noting that revealing detailed mechanisms of nanomaterials-cell interactions on cellular level has far going implications in the optimization of biomedical application potential of nanomedicines and minimization of their adverse effects [1, 13].

In recent years, a substantial body of research has demonstrated that mechanical forces play a pervasive role in living organisms and exert a direct influence on cell functions [17–20]. While the impact of mechanical forces on biology is most obvious during activities like breathing, heartbeats, blood circulation, and physical exercise, these forces also govern processes such as morphogenesis, cell migration, and cell adhesion to the extracellular matrix [17–22]. Crucially, it has become evident that mechanical forces can regulate a wide array of biological phenomena, spanning from cell growth and specialization to maintaining tissue equilibrium and orchestrating complex inflammatory reactions [18, 21–24]. Recent progress in mechanobiology has demonstrated that mechanical signals influence various cellular responses, with the extent of modulation varying depending on the cell type. Specifically, it has been demonstrated by a number of studies that substrate stiffness plays a crucial role as a regulatory factor in processes such as cell spreading, locomotion, differentiation, and proliferation. Therefore, it is not surprising that stiffness-regulated cell responses have been linked with modulation of nanoparticle uptake kinetics and processing by cells.

Although much is known separately about cell responses to their local physical environments and the size/shape dependent cellular uptake of different nanomaterials, the effects of the extracellular matrix (ECM) stiffness on cellular uptake and processing of nanoparticles generally are still poorly understood [25, 26]. It has been shown that matrix stiffness affects the cellular uptake of succinate micelles, silica and polystyrene nanoparticles [25–27]. It becomes evident that cell responses regulated by stiffness may lead to the changes in the nanoparticle uptake kinetics. Such effects may be used to develop and design novel nanoparticle formulations with improved targeting and delivery properties [26]. However, the influence of localized physical forces on the internalization of nanoparticles by cells remains largely unexplored. Importantly, systematic analysis of how mechanical cues may bias interactions of nanoparticles with cells is lacking in the literature. Important implication of such research is that the differences in cell-nanoparticle interactions between in vitro and in vivo settings can be attributed to the fact that cell-nanoparticle interactions are not solely determined by the physicochemical properties of the particles [27]. It has been illustrated that cultivating cells on substrates of varying stiffness results in distinct levels of particle uptake. Stiffer substrates were found to enhance spreading and internalization of nanoparticles [26]. Given that cells can respond differently to stiffness signals, it is essential to characterize their influence on uptake for the enhancement of nanoparticle-based therapeutic approaches. Additionally, studies revealing how substrate stiffness impacts the cellular uptake of nanoparticles holds significant implications in the other aspects. For instance, in physiological conditions, tumor tissues exhibit diverse stiffness levels across different stages, and metastatic cancer cells migrate through tissues with varying stiffness [28–31]. If the mechanical properties of ECM do indeed regulate cellular uptake, such effects must be considered in optimizing nanoparticle-based targeting of cancer cells to inhibit tumor growth and cancer cell metastasis.

Recent studies tried to address those challenging questions of how mechanical forces may bias nanoparticle-cell interactions [25–27]. In fact, some basic results and conclusion were achieved. Therefore, here we will review and critically analyze evidence of the impact of mechanical cues on cell-nanoparticle interactions. We will describe current in vitro experimental platforms utilized to study effects of mechanical forces on cell-nanoparticle interactions. We will provide systematic analysis of current state of the art in our understanding on how and to which extent mechanical cues affect cell-nanoparticle interactions. In this review we would like to focus on potential mechanisms mechanical regulation of nanoparticle uptake and processing by cells. We will also discuss challenges and perspectives in this research direction. Systematic analysis that we would like to provide in this review could be utilized as a new avenue to optimize nanoparticle designs for more effective in vivo delivery and will provide a foundation for future therapeutic optimization of nano-based medicines.

## Mechanical cues regulate cell fate

**Fig. 1:**
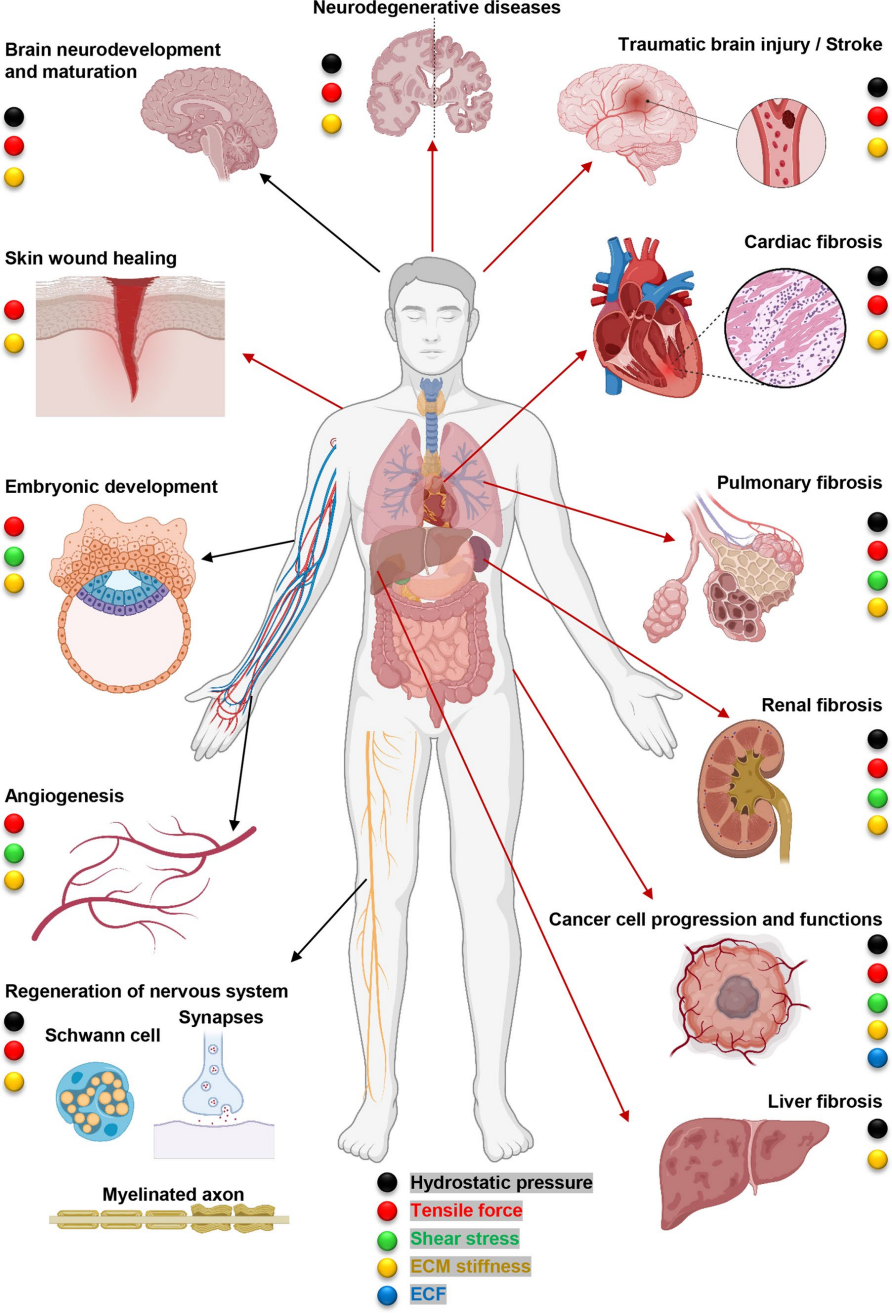
Schematic overview of impact of mechanical cues on different physiological (black arrows) and pathophysiological (red arrows) processes. Mechanical cues play an important role in physiological processes, such as embryonic development, skin and wound repair, angiogenesis and vascular remodeling, and nervous system regeneration, neurodevelopment and maturation of the nervous system. Emerging evidence suggests that mechanical cues are crucial regulators of pathophysiological processes associated with various diseases, such as pulmonary fibrosis, neurodegenerative diseases, cardiac fibrosis, renal fibrosis, liver fibrosis, and cancer progression. This figure was inspired by open-access article ref. [24]. ECM, extracellular matrix; ECF, extracellular fluid viscosity. Created with BioRender.com

Within the microenvironment of tissues, cells experience a diverse array of physical and chemical cues [18, 32]. The surrounding tissue environment serves as a dynamic area where cells constantly interact with various molecular and mechanical factors, shaping their responses and contributing to the overall physiological processes. This interplay of signals within the tissue microenvironment underscores the complex and multifaceted nature of cellular regulation and function [18, 32]. The structure and composition of the cellular microenvironment have been the subject of extensive investigation due to its role as a key regulator of both cell fate and behavior [19, 24, 33–37]. Equally significant in understanding cellular dynamics is the consideration of mechanical forces exerted on cells. These mechanical forces, such as tension, compression, and shear, contribute substantially to the overall cellular regulatory framework. Therefore, a comprehensive understanding of cellular function requires a holistic approach that combines both biochemical compositions and the mechanical forces within the microenvironment. Additionally, various physical factors have been associated with numerous pathophysiological processes [24, 30]. Indeed, pathophysiological processes, where a variety of mechanical cues plays crucial role, span from embryonic development and tissue repair to wound healing, neural regeneration, neurodevelopment and maturation of the nervous system, neurodegenerative diseases, fibrosis, tumorigenesis, cancer progression and even the resistance mechanisms observed in cancer immunotherapy (Fig. 1) [24, 38–43]. Thus, unraveling the molecular mechanisms of cellular mechanotransduction will bring insights contributing to advancements in fields such as regenerative medicine, oncology, and immunotherapy. Before we will analyze impact of mechanical cues on nanoparticle uptake and processing by cells, we need to briefly summarize what types of physical factors are known and what their role in physiological and pathophysiological processes.

*Hydrostatic pressure* (HP) is a prevalent force found within tissues and organs containing fluids, including blood vessels, the heart, eyes, joint cavities, and the urinary bladder. In hollow organs, various mechanical cues contribute to their dynamic environment, with hydrostatic pressure, sinusoidal stress, and interstitial fluid pressure being major factors. These forces collectively shape the mechanical landscape of hollow organs, influencing their physiological responses and contributing to the overall biomechanics of the surrounding tissues [24, 44]. HP in the interstitial cavity is normally negative approximately − 400 Pa [45]. Contrary in solid tumors and edematous tissues HP raises up to the range of 1–5 kPa [24, 30, 45]. HP within the normal physiological range plays a constructive role by fostering the development and repair of tissues. Research indicates that maintaining HP within this range actively promotes bone growth and organization in developmental models [46, 47].

It is worth noting that, when HP exceeds the normal range, categorized as pathological HP, it has the potential to induce decompensated lesions in tissues and organs, e.g. bladder fibrosis [24]. Additionally, HP can alter the conformation of ion channels and modulate ion transmembrane transportation, thereby influencing various pathophysiological processes. For instance, HP triggers the opening of transmembrane channels in HeLa cells, leading to an influx of calcium ions [48]. This process involves Piezo1, a mechanosensitive ion channel protein, which, upon exposure to HP, initiates the activation of mitogen-activated protein kinases (MAPK) and p38 signaling pathways [49]. Consequently, Piezo1 facilitates the expression of bone morphogenetic protein 2 (BMP2), influencing the phenotype of mesenchymal stem cells (MSCs) [49]. Moreover, elevated levels HP, particularly at 5.3 kPa, contribute to atrial electrophysiological remodeling and trigger an inflammatory response. This occurs through the regulation of ion flow, ultimately reducing atrial fibrillation [50]. The complex interplay of HP with ion channels and signaling pathways underscores its significant impact on cellular behavior and physiological responses [24].

HP signals propagate through diverse functional proteins and signaling pathways. In parenchymal cells like hepatocytes and hepatic stellate cells (HSCs), subcapsular HP influences biochemical processes [51]. Notably, cytoskeleton-related signals such as RhoA, ROCK, and α-SMA are activated by a 6.6 kPa HP stimulus on HSCs [52]. This elevation in interstitial fluid pressure activates HSCs, contributing to the progression of fibrosis [52]. In the context of hollow organs like the urinary bladder, the uroplakins (Ia/Ib/II/III) on the epithelial cell membrane play a crucial role in cell differentiation and constitute the primary components of the high resistance barrier in urinary bladder urothelium [24, 53, 54]. Sustained intravesical HP exceeding 4 kPa is generally recognized as pathological pressure, potentially leading to fibrosis [55]. Research indicates that HP reaching 19.6 kPa can induce the expression of uroplakin Ia and uroplakin II proteins in urothelial cells [56]. These proteins are key factors in activating the extracellular signal-related kinase (ERK) 1/2 pathway, emphasizing the intricate relationship between hydrostatic pressure and the molecular mechanisms governing cell behavior and tissue responses [56].

*Fluid shear stress* (FSS) arises within organs or capsules due to fluid flow. Human vasculature, including vessel bifurcations, stenosis, aortic aneurysms, heart valves, and capillary networks, experiences typical FSS in the form of shear and extensional flow [57]. Due to the lumen's structural characteristics the shear flow can be ranked as laminar or turbulent flow. Uniaxial extensional (elongational) flow involves flow acceleration parallel to the vascular wall, commonly occurring in regions with sudden contractions or expansions of fluid flow [24]. Stable or laminar flow contributes to anti-inflammatory, anti-adhesive, and anti-thrombotic effects on the vascular wall [58]. In contrast, persistent turbulent flow within the vascular wall can enhance endothelial permeability, leading to alterations in junctional proteins, and trigger proinflammatory signaling [59, 60]. This includes activation of NF-κB signaling and adhesion molecules, ultimately promoting the formation of lesions [59, 60]. The complex interplay between fluid shear stress and vascular dynamics underscores its significant role in regulating inflammation and vascular health [24].

Of note, FSS plays a crucial role in maintaining tissue homeostasis in various organs, including blood vessels, the heart, the airway, and the urinary bladder [24, 61, 62]. Elevated FSS has been associated with anti-inflammatory effects, such as the activation of KLF transcription factor 2/4 (Klf2/4) or endothelial nitric oxide synthase (eNOS) [24]. Conversely, turbulent flow, oscillatory patterns, and low FSS can trigger pro-inflammatory reactions [24]. It is worth noting that, FSS was found to be approximately 10 dyn/cm^2^ in large blood vessels whereas in arterioles and capillaries FSS is about 40–50 dyn/cm^2^ [63]. Numerous studies have demonstrated that turbulent flow or low FSS conditions (≤ 5 dyn/cm^2^) influence epithelial cells and play an important role in atherosclerosis, lipid deposition, and vessel wall thickening [64, 65]. Various signaling pathways in different vascular wall cells are altered by hemodynamic changes induced by blood flow [66–68]. Signaling pathways associated with FSS include vascular endothelial growth factor (VEGF), Notch, PDGF, Klf2, eNOS, endothelin, Rho family signaling molecules of the TGFβ/BMP/Smad pathway, MAPK signaling pathway, NF-κB signaling pathway, and GTPase signaling pathway [69, 70].

Cells respond to FSS mechanical signals through various mechanosensors, such as ion channels (K^+^, Ca^2+^), integrins, primary cilia, the glycocalyx, and G-protein-coupled receptors (for review see [24] and references therein). Additionally, Piezo channels, crucial sensors for mechanical stimulation, sense shear stress and transmit biomechanical signals to the nucleus, resulting in nuclear contraction [71]. Mechanosensory complexes, including VE-cadherin, VEGF receptor 2 (VEGFR2), and platelet endothelial cell adhesion molecule (PECAM-1 or CD31), are activated in response to FSS, transmitting biomechanical signals into endothelial cells [72]. It was shown that PECAM-1 interacts with type III intermediate filament subsequently activating Src via VE-cadherin support [73]. Further, G-protein-coupled receptors are eliciting an activation of Ras and Rho GTPase signaling cascades, influencing endothelial cell migration and traction force modulation [74, 75]. VEGFR2 may also induce activation of PI3K/AKT/mammalian target of rapamycin (mTOR) signaling axis which leads to integrin activation [76].

*Tensile force* (TF) or alternatively stretching force, plays a pivotal role in various physiological processes such as muscle and joint movement, atherogenesis, cardiovascular remodeling, and important cellular functions such as proliferation, transformation, and development [24, 77]. For example, the dynamic tension within joints can significantly influence the ultimate behaviors of muscles [78]. TF generated by blood flow, in the cardiovascular system, affects cellular behavior of multiple cell lineages like endothelial cells, smooth muscle cells, and cardiac myocytes [24]. Indeed, the flow of blood within the body creates mechanical pressures, including shear stress and circumferential stretch, which directly impact the endothelium [79]. The pressure exerted radially generates an internal circumferential stress within the vessel wall, while the stretching force longitudinally produces an internal longitudinal stress. Furthermore, a secondary tensile force emerges in the longitudinal direction due to the tethering of the vessel to surrounding tissue at its ends and various points along its length [79]. TF is known to induce cardiomyocyte hypertrophy by activating nuclear factor-like 2 (Nrf2) and interferon-regulated transcription factors in myocardial tissue [80]. Furthermore, vascular remodeling and contraction is supported by TF excreted in the vascular wall [81]. TF also triggers urothelium proliferation through α6-focal adhesion kinase (FAK) signaling and plays a role in the development of animal neurons via gene transcription regulation [82–84].

**Fig. 2:**
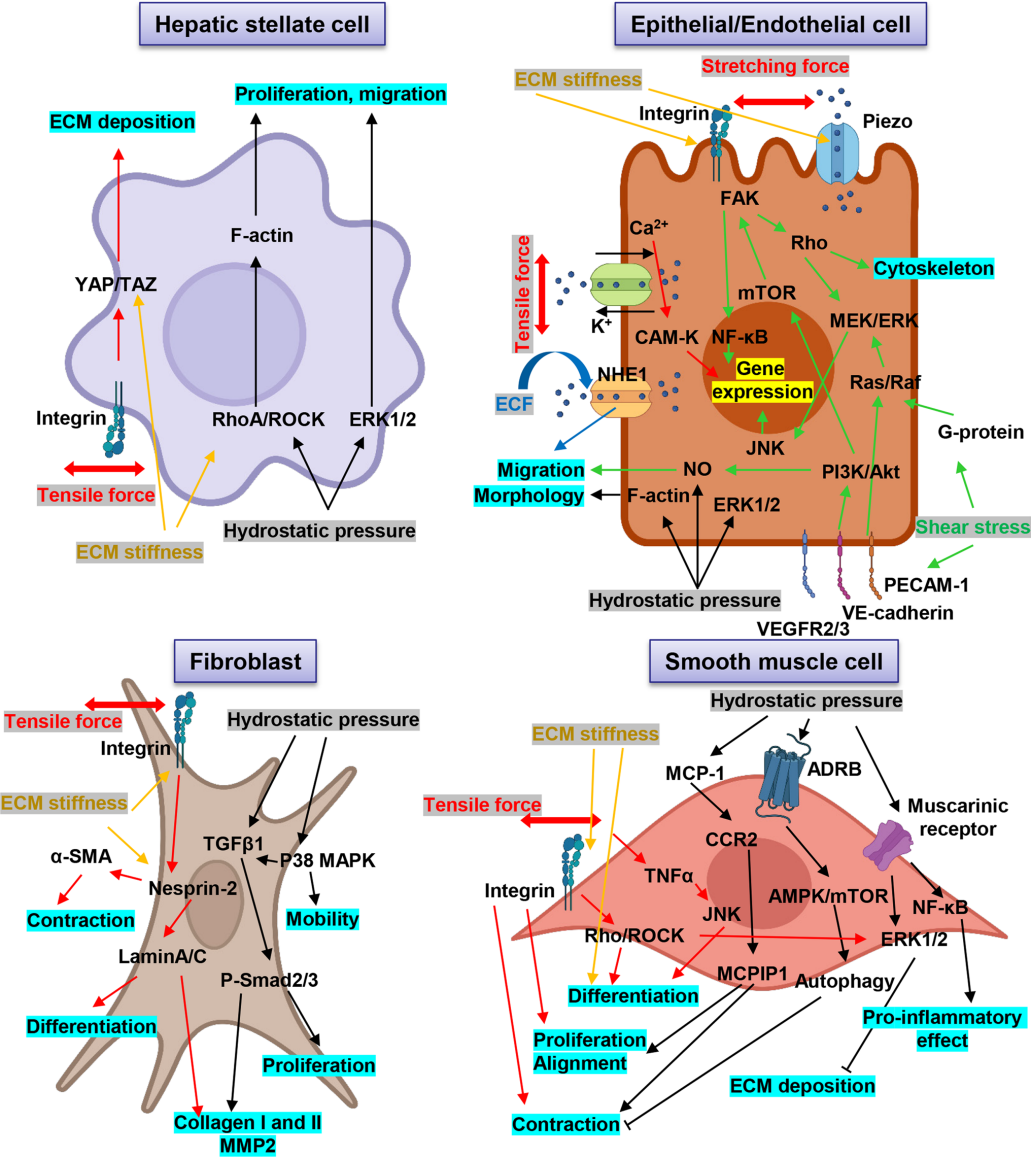
Schematic representation of molecular mechanisms how different mechanical cues (tensile force, extracellular fluid viscosity, hydrostatic pressure, and shear stress) regulate functions of different cell types. Red arrows indicate stretching/tensile forces, green arrows indicate shear stress, blue arrows indicate impact of extracellular fluid viscosity, and black arrows refer to hydrostatic pressure, yellow arrows indicate effect of ECM stiffness. ADRB, β adrenoceptors; ECM, extracellular matrix; ERK, extracellular signal-related kinase; MCP1, monocyte chemotactic protein 1; MCPIP1, MCP1-induced protein; MMP, matrix metalloprotease; mTOR, mammalian target of rapamycin; NF-κB, nuclear factor-κB; TGFβ, transforming growth factor β; TNFα, tumor necrosis factor α; VCAM-1, vascular cell adhesion molecule 1; ECF, extracellular fluid viscosity; NHE1, Na^+^/H^+^ exchanger-1; PECAM-1, platelet and endothelial cell adhesion molecule 1; VEGFR2/3, vascular endothelial growth factor receptor 1; FAK, focal adhesion kinase; MEK, mitogen-activated protein kinase kinase; ERK, extracellular signal-regulated kinase; MAPK, mitogen-activated protein kinase; Akt, AKT serine/threonine kinase; PI3K, phosphoinositide 3-kinase; ROCK, Rho-associated protein kinase; YAP, yes-associated protein; TAZ, tafazzin; phospholipid-lysophospholipid transacylase; CCR2, C–C chemokine receptor type 2; AMPK, AMP-activated protein kinase. Created with BioRender.com

Parameters of TF, like magnitude, frequency, and duration, greatly affect the extent and amplitude of physiological processes being regulated by TF [24]. In fact, TF was found to significantly affect the alignment, differentiation, migration, proliferation, survival, apoptosis, and autocrine and paracrine functions of cells (Fig. 2) [24]. Upon receiving biomechanical signals, integrins transduce these signals into cells, leading to the activation of p38MAPK signaling, nitric oxide (NO), and reactive oxygen species (ROS), which, in turn, trigger downstream cascades [85, 86]. Concurrently, active JNK and p38MAPK signaling pathways lead to the elevated expression of α-smooth muscle actin (α-SMA) and promoter activities [87]. Additionally, the ERK signaling pathway is upregulated via activation of Rho by integrins [88]. Ultimately, these molecular events regulate the phenotype and alignment of cells [24].

*The stiffness of ECM.* ECM is a non-cellular component found in extracellular capsules. It is attributed to a complex composition primarily consisting of collagen, fibronectin, elastin, lamin, proteoglycan, glycoprotein, and glycosaminoglycan [89]. Fibrillar proteins, notably collagens, exhibit high tensile strength but low elasticity, whereas elastic fibers, such as elastin, display high elasticity but low tensile strength. Consequently, the constituents of the ECM significantly influence the mechanical properties of tissues [90]. Primarily, ECM reorganization dramatically affects and regulates cell growth, differentiation, and apoptosis [24]. The intracellular skeleton and cell surface adhesion molecules mediate cellular communication through the interaction with both the ECM and neighboring cells [24]. Of note, ECM remodeling is tightly linked with complex physiological conditions, and pathological ECM exerts a lasting impact on the morphology and functions of cells [91]. It amplifies the ECM deposition process, leading to severe fibrosis [91].

It is worth noting that excessive ECM deposition can have detrimental consequences. Indeed, myocardial fibrosis arises from fibrotic scarring due to myocardial infarction, leading to matrix deposition in interstitial and perivascular areas. This condition impairs heart function and supports the progression of heart failure [92]. Emerging evidence suggests that predominant increase in type I and type III collagen fibers in hypertensive heart disease and heart failure is associated with aortic stenosis [93, 94]. Additionally, liver fibrosis is triggered by myofibroblasts resulting from chronic hepatotoxic injuries, such as hepatitis B or C, alcohol abuse, or cholestatic injury (for example, bile duct obstruction) [95–98]. Of note, liver fibrosis can be also triggered by venostasis in patients with right ventricular heart failure which can be caused by hypoxia and/or increased HP [99]. The ECM remodeled by hepatic stellate cells promotes the transformation from fibroblasts to myofibroblasts, ultimately replacing normal liver tissues with cross-linked type I and type III collagen fibers [95–98].

In fact, various biological processes are modulated by the mechanical properties of ECM. The stiffness of the ECM serves as an indicator of tissue resistance to deformation, influencing an increase in tissue elasticity [17, 33, 89, 98]. ECM stiffness is regulated not only by qualitative and quantitate composition of the ECM but also by post-translational modifications of ECM components. For example, the elasticity of the ECM can be increased by nonenzymatic glycation and cross-linking of collagen [17, 33, 89, 98]. ECM stiffness exhibits variations across different organs. Additionally, ECM stiffness changes during development of different pathophysiological conditions. To illustrate, normal lung tissues typically have a Young’s modulus of approximately 1 kPa, essential for respiratory function [100]. However, in patients with idiopathic pulmonary fibrosis, enhanced ECM deposition and the transformation of contractile myofibroblasts leads to significant increase in lung elasticity up to 30–50 kPa [101]. Similarly, the stiffness of normal liver tissue ranges from 1.5–4.5 kPa, while in the early and late stages of liver fibrosis, it is elevated up to 4.1–12.9 kPa and 16.3–48 kPa, respectively [102–107]. These variations highlight the dynamic nature of tissue mechanics in different physiological and pathological conditions. It is worth noting that liver stiffness organization in vivo is more complicated than simple state of the tissue and ECM. Clinical hepatology defines liver stiffness as a sum of fluid pressure and mechanical stiffness of the tissue [108, 109]. In fact, portal hypertension is a major contributor to the overall stiffness of the liver [108, 109].

Although molecular mechanisms of how ECM stiffness modulates cellular function are still not fully deciphered, substantial progress has been made in last years. Indeed, increased ECM stiffness promotes cell–cell communication by activating integrins [24]. The integrins, predominantly present in fibroblasts, play a pivotal role in receiving signals from ECM components, transducing both mechanical and biochemical cues into cells. This transduction facilitates various cellular processes, including proliferation, differentiation, migration, and invasion [110–113]. The integrin activation results into execution of the RhoA/ROCK pathway, which leads to increased accumulation of collagen and fibronectin [114]. These events trigger activation of the transcriptional regulator BTBD7, that subsequently initiates epithelial-to-mesenchymal transition (EMT) through Snail2, inhibiting E-cadherin, and weakening cell adhesion [115]. Furthermore, intracellular cytoskeletal protein talin interacts with the cytoplasmic tail of β integrin. This interaction results into F-actin assembly and boosts signal transduction [111, 116, 117]. Additionally, the integrins interact with FAK leading to activation of downstream signaling that transforms fibroblast phenotype [118, 119]. The actin-myosin II binding transduces biomechanical signals to the nucleus, translocating Yes-associated protein (YAP) and transcriptional coactivator with PDZ-binding motif (TAZ) into the nucleus [120–123]. This triggers transcription upregulation of downstream genes that regulate collagen synthesis, cell proliferation, and cell differentiation. This events consequently increase ECM stiffness [120–123]. Notably, ECM stiffness-induced transduction of biochemical stimuli, that is dependent on YAP/TAZ activity, is mediated by multiple signaling pathways, including TGFβ, Wnt/β-catenin, MAPK/ERK, and NF-κB [124–126].

*Extracellular fluid (ECF) viscosity.* Recently, novel mechanical factor, namely ECF viscosity, that affects cell behavior and function, was identified [127]. It was shown that ECF viscosity may interact with ECM stiffness, influencing cell migration and substrate mechanotransduction [127]. Important fluid parameters, such as density, osmotic pressure, and viscosity are regulated by the abundance of macromolecules presented in ECF [24]. Logical expectation is that high ECF viscosity might intuitively hinder the motility of various cell types. However, it was found unexpectedly that increased ECF viscosity actually facilitates cell migration and cancer dissemination [127]. The interplay between ECF and cells initiates the formation of an actin-related protein 2/3 (ARP2/3)-complex-dependent actin network, leading to the polarization of the Na^+^/H^+^ exchanger-1 (NHE1) [127]. NHE1, an isoform of the Na^+^/H^+^ exchanger, acts as a membrane transporter, exchanging intracellular protons for extracellular sodium ions [128]. The activation of NHE1 induces cell swelling, elevates cell tension, and promotes TRPV4-mediated calcium influx. Consequently, cell motility is increased through activation of RhoA-dependent contractility [127]. High ECF viscosity has been observed to boost cell motility on two-dimensional substrates. Moreover, it has been confirmed that ECF viscosity promotes dynamic cell spreading acting via integrin/YAP signaling [129]. The elevated viscosity of ECF promotes integrin-based adhesion, thereby augmenting cell migration. Importantly, ECF can also synergize with ECM stiffness to enhance cellular mechanotransduction [24]. Despite these findings, the precise mechanisms underlying the effect of ECF viscosity on cells remain poorly understood.

## Models of physical stimuli used in nanobio research

From chapters above one can clearly see that mechanical cue of cellular microenvironment dramatically affect cell fate and even regulate crucial cellular functions. Therefore, it is logical to assume that such rewiring of cellular activity might be possibly translated towards nanoparticle processing by cells. While there is considerable individual knowledge regarding how cells respond to their specific physical surroundings and the size/shape-dependent uptake of various nanomaterials, the impact of physical factors on the overall cellular uptake and processing of nanoparticles remains fragmented and inadequately understood. This knowledge gap highlights the need for further research and a more cohesive exploration of the complex interplay between cellular responses, ECM properties, and nanoparticle dynamics. However, emerging studies shed some light on this complicated problem. For instance, studies indicate that ECM stiffness modulates cell-nanoparticle interactions and uptake of different nanomaterials, such as succinate micelles, silica and polystyrene nanoparticles [25–27]. Therefore, further we would like to summarize current knowledge on modulation of cell-nanoparticle interactions by mechanical cues. It is worth noting here that for a comprehensive understanding of how ECM stiffness specifically influences the uptake and processing of nanoparticles, we need first to summarize the current experimental platforms employed for evaluating the impact of physical stimuli on interactions between cells and nanoparticles.

We summarized main current studies and experimental models utilized in the research of physical stimuli impact on nanoparticle processing by cells in Table 1. To the best of our knowledge we could not identify any study dealing with the effects of extracellular fluid viscosity on nanoparticle-cell interactions (Table 1). This is not surprising due the fact that the ECF viscosity effects on cell behavior and function were described relatively recently [127]. One can clearly see that the most studied physical factors are ECM stiffness and shear stress (Table 1). It is understandable due to the availability of custom-made and commercial platforms that enable application of those cues. To replicate ECM stiffness, researchers commonly employ polydimethylsiloxane (PDMS), or polyacrylamide gel substrates coated with collagen or fibronectin, as indicated in Table 1. It is worth noting, that currently a wide array of experimental models exists for modulating substrate stiffness, ranging from synthetic polymers to decellularized ECM sourced from animals, for review see [24, 130–132] and references therein. Furthermore, the elastic modulus can be adjusted dramatically, spanning orders of magnitude (e.g., 1–100 kPa) to suit the specific requirements of the study [24, 130–132]. Additionally, tightly controlled three-dimensional (3D) matrices are available for mimicking the ex vivo tissue microenvironment (refer to [24, 130–132] for detailed information). Despite this, studies primarily focusing on the impacts of mechanical cues predominantly use 2D cell culture systems, as outlined in Table 1. Even though 2D systems offer a relatively inexpensive and straightforward means of simulating ECM mechanical cues, 3D systems provide a superior microenvironment mimicking in vivo conditions [24, 133].

**Table 1:** Experimental models utilized in the research of physical stimuli impact on nanoparticle processing by cells

Mechanical cue	Experimental models	Characterization	References
ECM stiffness	Fibronectin-coated polyacrylamide gel substrates	Young’s modulus: 1.61 ± 0.11 kPa; 3.81 ± 0.12 kPa; 5.71 ± 0.51 kPa	[26]
ECM stiffness	Collagen I-coated polyacrylamide gel substrates	Young´s modulus: 1 ± 0.2 kPa; 7 ± 0.7 kPa; 20 ± 1.3 kPa; 25 ± 1.5 kPa	[25]
ECM stiffness	TCPS; fibronectin-coated PDMS substrates	Young´s modulus: GPa (TCPS); 2.8 MPa (PDMS); 130 kPa (PDMS)	[27]
ECM stiffness	Collagen I-coated polyacrylamide gel substrates	Young´s modulus: 0.25 ± 0.05 kPa; 10.5 ± 0.02 kPa	[176]
ECM stiffness	Fibronectin-coated PDMS substrates	Young´s modulus: 0.39 ± 0.03 kPa; 4.5 ± 0.5 kPa; 51.4 ± 4.5 kPa	[179]
ECM stiffness	Fibronectin-coated polyacrylamide gel substrates	Young´s modulus: 1.0 ± 0.3 kPa; 4.0 ± 0.8 kPa; 10.0 ± 1.6 kPa; 20.0 ± 1.2 kPa; 40.4 ± 2.4 kPa	[177]
ECM stiffness	Collagen I-coated polyacrylamide gel substrates	Young´s modulus: 1 kPa; 9 kPa; 31 kPa	[213]
ECM stiffness	Collagen I-coated commercial silicone surfaces (CytoSoft®)	Young´s modulus: 0,5 kPa; 2 kPa; 8 kPa; 16 kPa; 32 kPa; 64 kPa	[213]
ECM stiffness	Collagen I-coated polyacrylamide gel substrates	Young´s modulus: 3 kPa; 30 kPa	[178]
Shear stress	Piezoelectric pressure controller (model OB1 Mk3, Elveflow)	Flow rate of 22.89 μl/min; Shear stress of 0.025 dyne/cm^2^	[183]
Shear stress	Custom-made microfluidic chip connected to a peristaltic pump and media reservoir	Flow rate from 1.5 to 5.0 ml/min; Shear stresses from 2 to 8 dyne/cm^2^	[187]
Shear stress	Rhombic chamber chips from microfluidic ChipShop (Jena, Germany)	Shear stress of 0.7, 3.0, 6.0 and 10.0 dyne/cm^2^	[184]
Shear stress	A peristaltic pump (Watson and Marlow, UK) furnished with silicon tubes (internal diameter 1.6 mm, total length 250 mm)	Flow speed ~ 50 cm/s	[188]
Shear stress	Custom-made chip containing six microchannels with a height of 100 μm and width of 1 mm each connected to a multichannel peristaltic pump	Flow rate of 0.907 μl/min; Shear stress of 10 dyne/cm^2^	[185]
Shear stress	VenaFlux™ Platform (Cellix Ltd)	Shear stress from 0.1 to 10 dynes/cm^2^	[189]
Shear stress	Custom-made syringe pump	0.0146 dyne/cm^2^ 0.146 dyne/cm^2^	[186]
Tensile forces	Flexercell® Tension Plus™ System (Flexcell Int.)	10% cyclic uniaxial tensile strain at a frequency of 1 Hz	[193]
Tensile forces	Flexcell® tension system (Flexcell Int.)	Cyclic stretch with frequency of 1 Hz and cyclic strain (5%, 10%, and 15%)	[214]
Tensile forces	Flexcell® tension system (Flexcell Int.)	Cyclic stretch with frequency of 1 Hz and 10% equibiaxial strain	[192]
Tensile forces	Flexcell® tension system (Flexcell Int.)	Cyclic stretch with frequency of 1 Hz and 5% cyclic elongation	[208]
Interstitial fluid pressure	Xenograft tumors in nude mice implanted via subcutaneous injection of 1 × 10^6^ cells in 50% Matrigel	~ 10–20 mmHg	[194]

TCPS, standard tissue culture polystyrene; PDMS, polydimethylsiloxane

**Fig. 3:**
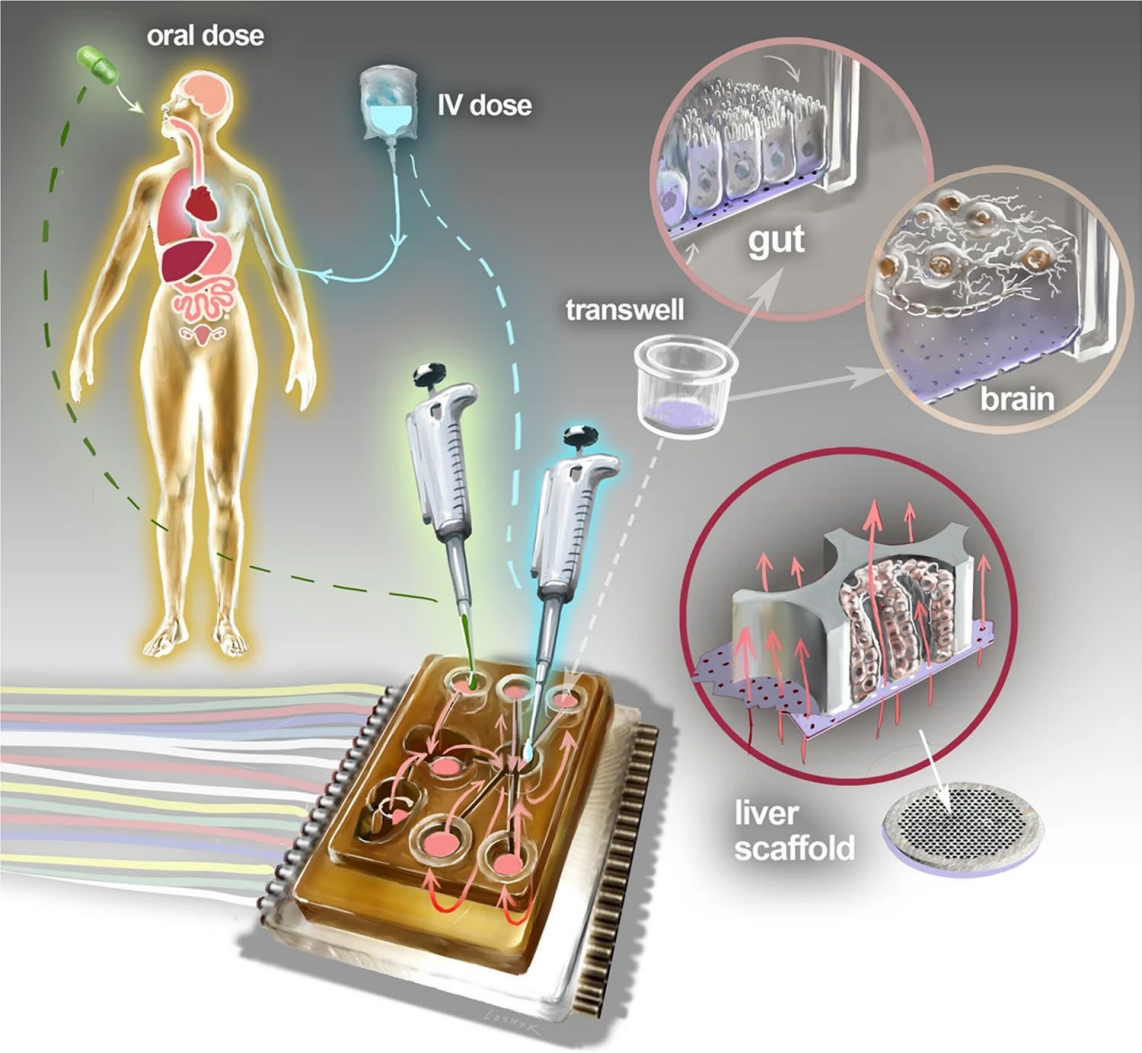
Schematic overview of Physiome-on-a-chip approach. The Physiome-on-a-Chip comprises bioengineered devices that nurture many interconnected 3D MPSs representing specified functional behaviors of each organ of interest, designed to capture essential features of in vivo physiology based on quantitative systems models tailored for individual applications such as drug fate or disease modeling. By interconnecting MPSs, dynamic multi-organ signaling can be recreated naturally through cytokine and hormone circulation, cell trafficking, and metabolic byproducts. Multi-MPS systems bridge the complexity gap between traditional in-vitro cell culture, animal models, and human patient samples, potentially providing better prediction of human responses at lower financial and ethical costs as compared to current methods of drug development. Illustration by Victor O. Leshyk. Reprinted from open-access article ref. [211] under the terms of the Creative Commons CC BY license

**Fig. 4:**
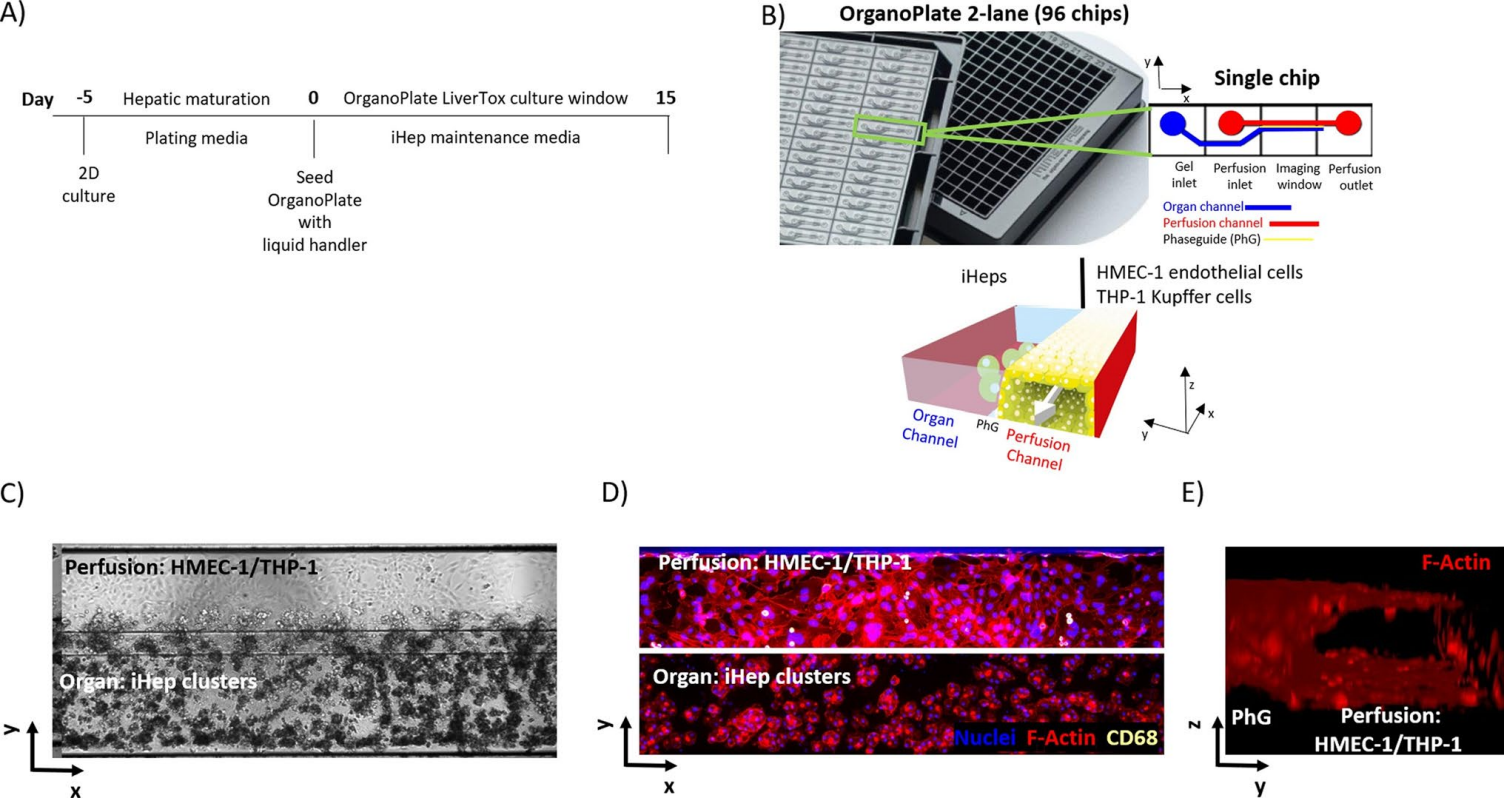
OrganoPlate LiverTox culture set up. **A** Plating media were used to differentiate iHeps in 6 well microtiter plates in 2D culture for 5 days prior to Day 0 harvest and seeding by liquid handler as 3D aggregates in the OrganoPlate 2-lane. The OrganoPlate LiverTox model was cultured in iHep maintenance media and assayed for up to 15 days post seeding. **B** Image of the OrganoPlate 2-lane 96 chips and schematics of the perfusion and organ channels separated by a Phaseguide (PhG). **C** Phase contrast image of the fully assembled 3-cell type liver-on-a-chip showing iHep clusters in the organ channel and HMEC-1 endothelial and differentiated THP-1 Kupffer cells in the perfusion channel. **D** Fluorescent maximum intensity projection demonstrating nuclei/F-actin/CD68 in blue/red/yellow. **E** 3D reconstruction of perfusion channel demonstrating the presence of a 3D tubule structure. Reprinted from open-access article ref. [212] under the terms of the Creative Commons CC BY license

Current advances in microfluidics, tissue engineering and microfabrication led to the development of sophisticated platforms, including organ-on-chip (OoC) systems, to study effect of shear stress and hydrostatic pressure on living cells [130, 132, 134–136]. Indeed, by integrating developments in tissue engineering and microfabrication, OoC platforms have emerged as a cutting-edge experimental tool for delving into human pathophysiology and comprehending the impact of therapeutic interventions within the human body [134–136]. These platforms represent a next-generation approach, showcasing a convergence of innovative techniques that enable more precise and physiologically relevant investigations compared to traditional experimental methods. The fusion of tissue engineering and microfabrication techniques in OoCs has garnered considerable attention, positioning them at the forefront of scientific inquiry for unraveling complicated aspects of human physiology and assessing the efficacy of therapeutic strategies (see examples on Figs. 3 and 4) [134–136]. This combination not only enhances our understanding of pathophysiological processes but also provides a sophisticated means to evaluate the effects of various treatments, paving the way for more informed and targeted medical interventions [134–136]. However, when it comes to the study effects of shear stress and hydrostatic pressure on nanoparticle-cell interactions, relatively simple commercial systems are abundantly used (Table 1).

It is worth noting that OoC technology is steadily progressing toward widespread acceptance as a dedicated experimental platform tailored for human-centric preclinical research and therapeutic testing [134–136]. Current studies are progressively demonstrating a higher degree of predictiveness, closely aligning with clinical data, and reflecting the integrated complexities of human physiology [134–136]. Therefore, there are plenty commercially available sophisticated platforms for drug testing in the pharmaceutical industry including large pharmaceutical companies like GSK, Roche and Pfizer [137–139]. So far, studies on effects of mechanical cues on nanoparticle-cell interactions utilizing those sophisticated systems are underrepresented (Table 1).

It's essential to emphasize that the physical properties of nanoparticles are significant factors in regulating their interaction with cells [140, 141]. Specifically, parameters such as nanoparticle shape and stiffness are crucial for governing uptake, particularly in the context of external mechanical influences. Therefore, in the upcoming chapters, we will provide a brief overview of how nanoparticle shape and stiffness impact their interaction with living cells.

## Influence of nanoparticle shape on interaction with cells

Advancements in nanotechnology have facilitated the design and fabrication of nanoparticles in a myriad of shapes tailored to specific requirements, including spheres, ellipsoids, rods, and cones [140–143]. It is well documented that the shape of the nanoparticle plays a pivotal role in the uptake pathway as well as trafficking within cells [140–143]. Nanoparticle shapes are typically classified into various dimensions: zero-dimensional (0D) for nanoparticles, one-dimensional (1D) for structures like nanorods, nanofibers, nanopillars, and nanowires, two-dimensional (2D) for nanosheets, nanoplates, and nanopores, and three-dimensional (3D) for nanocomposites and intricate hierarchical structures [144].

The relationship between nanoparticle shape and cell internalization mechanisms is a multifaceted phenomenon. Several shape- and size-dependent parameters have been identified as critical determinants in nanoparticle internalization [140–143]. These parameters include: (a) the surface-to-cell membrane contact area, which influences particle-cell adhesion, (b) the strain energy required for membrane deformation, and (c) sedimentation or local particle concentration at the cell membrane-particle interface [145]. These factors collectively influence the likelihood of nanoparticle internalization, which is constrained by factors such as shear stress and optimal nanoparticle size.

Research has demonstrated that nanoparticles with extremely low or high aspect ratios are not typically engulfed by endocytosis [146]. Instead, the aspect ratio should fall within a range favorable to complete wrapping of particles by the cell membrane [146]. Furthermore, it has been shown that oblate shapes adhere more effectively to biological substrates than classical spherical particles of equivalent volume, potentially enhancing therapeutic efficacy [147].

Molecular dynamic simulations revealed that spherocylindrical nanoparticles are more efficiently endocytosed than spherical nanoparticles, with cells unable to effectively uptake cylindrical particles due to their sharp ends. This discrepancy in nanoparticle shape-dependent endocytosis was attributed to variations in surface adhesion energy among different shapes [148]. These findings were corroborated by the research, demonstrating that disc-like negatively charged nanoparticles with high aspect ratios exhibit greater cellular uptake compared to nanorods and lower aspect-ratio nanodiscs in mammalian epithelial and immune cells [145]. It was proposed that less strain energy is required for bending of cell membranes around nanodiscs than around nanorods, resulting in higher cellular uptake [145]. Subsequently, it was found that the cellular uptake of nanoparticles varies depending on their shape, with spheres demonstrating the highest uptake, followed by cubes, rods, and discs [149]. This hierarchy in uptake is attributed to the ease of bending of the cell membrane around the particles. Interestingly, star-shaped nanoparticles were found to be rapidly enveloped by the cell membrane, similarly to their spherical counterparts, leading to increased uptake [149]. These insights collectively underscore the complex interplay between nanoparticle shape and cellular internalization mechanisms, highlighting the importance of shape optimization in nanomedicine applications.

Advancements in computational methods have facilitated the conduct of high-quality theoretical and simulation studies, enabling the exploration of the effects of nanoparticle shape on interactions with cells [150]. For instance, dissipative particle dynamics (DPD) simulations were applied to explore the processes of physical transfer of nanoparticles with various shapes, such as spheres, ellipsoids, rods, discs, and pushpin-like particles, across lipid bilayers [151]. The factors such as the contact area between the particle and the lipid bilayer and the local curvature of the particle at the contact point play crucial role in regulating nanoparticle penetration through lipid bilayer [150]. Notably, the efficacy of nanoparticle penetration across this multicomponent lipid layer was observed to follow a hierarchy: rods exhibited greater penetration than discs, which in turn surpassed spheres [152]. This suggests that nanoparticle shape influences their interaction with lipid bilayers, with elongated structures such as rods demonstrating enhanced penetration compared to their spherical counterparts [150].

Furthermore, studies have been conducted to examine the receptor-mediated endocytosis controlling uptake of nanoparticles with varied shapes. The coarse-grained molecular dynamics (CGMD) models were applied to systematically explore the receptor-mediated membrane wrapping of nanoparticles with diverse shapes [153]. It was found that the efficiency of nanoparticle membrane wrapping during endocytosis depends on the interplay between receptor diffusion kinetics and thermodynamic driving forces [153]. Receptor diffusion kinetics refers to the kinetics of receptor recruitment, influenced by the contact edge length between the nanoparticle and membrane. On the other hand, the thermodynamic driving force denotes the amount of free energy necessary to propel nanoparticle into cells [153]. For instance, oblate nanoparticles encounter a higher energy barrier compared to prolate nanoparticles. However, oblate nanoparticles boast a significantly larger contact edge length, facilitating faster wrapping than prolate nanoparticles [150, 153].

Additionally, endocytosis kinetics was assessed utilizing ligand-coated nanoparticles with different shapes, such as spherical, rod-shaped, and disc-shaped nanoparticles [154]. One can discriminate invagination and wrapping stages in the nanoparticle internalization processes that depends on the shape anisotropy of nanoparticles [154]. During the membrane invagination stage, facilitated by strong ligand-receptor binding energy, nanoparticles rotated to maximize their contact area with the membrane. Subsequently, the kinetics of the wrapping stage were primarily governed by membrane tension and nanoparticle area with the largest local mean curvature, where the membrane underwent the most pronounced bending [154]. Consequently, nanoparticles of varying shapes exhibited distinct favorable orientations for the two stages, potentially requiring one or more orientation rearrangements during the endocytosis process [154].

## Effects of nanoparticle stiffness on interaction with cells

The inherent stiffness of nanoparticles plays a critical role in determining their uptake by cells [142, 143, 150]. This stiffness refers to the nanoparticles' ability to resist deformation when subjected to external forces. Terms such as stiffness, hardness, elasticity and rigidity are interchangeably used to describe this property [142, 143, 150]. Young’s modulus, measured in Pascals (Pa), serves as a key indicator of nanoparticle elasticity. Nanoparticles with higher Young’s modulus values, termed hard nanoparticles, include gold nanoparticles, quantum dots, and magnetic NPs. In contrast, soft nanoparticles, characterized by lower Young’s modulus values, encompass materials like hydrogels, liposomes, and biodegradable polymers [142, 143, 150]. Understanding the elasticity of nanoparticles is crucial for predicting their interactions with biological systems.

Current evidence suggests that nanoparticle stiffness significantly impacts on cellular uptake, revealing a consistent trend wherein cells exhibit a preference for internalizing stiffer nanoparticles over softer counterparts [155, 156]. This preference is attributed to the reduced energy expenditure of cell membranes when engulfing stiffer nanoparticles, despite variations in the deformational energy required during the internalization process [142, 143, 150]. Moreover, computational modeling utilizing CGMD simulations supports these experimental findings, indicating shifts in deformational energy during the internalization of stiff and soft nanoparticles [157]. However, alternative hypothesis exists, with some studies suggesting that softer nanoparticles are internalized more efficiently [158, 159], while others propose intermediate stiffness as optimal for cellular uptake [160]. In fact, tuning nanoparticle stiffness could be utilized as drug delivery strategy, as it was shown by enhanced accumulation of nanolipogels within tumor cells through the manipulation of nanoparticle stiffness [161].

Modeling the influence of nanoparticle stiffness on cellular uptake presents distinct challenges compared to size and shape simulations [142, 143, 150]. Consequently, specialized techniques are requisite for accurate simulation. For instance, one study conducted comparative analysis between poly(lactic–co-glycolic acid) (PLGA)–lipid (P–L) nanoparticles and PLGA–water–lipid (P–W–L) nanoparticles, including liposomes, to elucidate the mechanisms underlying rigidity-mediated nanoparticle cellular uptake [162]. The obtained findings revealed distinct behaviors: rigid P–L nanoparticles were wrapped by the cell membrane and internalized smoothly, whereas less rigid P–W–L nanoparticles experienced substantial deformation during internalization, eventually becoming entrapped within the cell membrane [162]. This observation underscores the complexity of the cellular uptake process, particularly regarding the interplay between nanoparticle stiffness and membrane interactions.

Furthermore, soft spherical nanoparticles were found to exhibit markedly elevated energy barriers attributed to their deformability [153, 157]. Due to the elevated energetic barrier, the recruitment of a greater number of receptors was needed to facilitate membrane wrapping, providing the necessary force for membrane wrapping for cellular uptake of soft spherical nanoparticles [153, 157]. Consequently, soft spherical nanoparticles were observed to undergo incomplete wrapping in comparison to their rigid counterparts [153, 157].

## Effects of mechanical stimuli on nanoparticle uptake by cells

Emerging evidence suggest that cellular uptake of nanoparticles and their subsequent subcellular localization are biased when subjected to specific physical stimuli [25–27]. Although current body of knowledge regarding the processes by which cells internalize nanoparticles and how the application of various mechanical cues influences this crucial aspect is fragmented, it is important to summarize recent findings to provide insights into the fundamental processes at play and highlight potential avenues for further investigation, thereby advancing fundamental understanding of the interplay between mechanical cues and cellular responses to nanoparticles.

We summarized the main current findings on effects of mechanical cues on nanoparticle-cell interactions in Table 2. The first conclusion what can be derived from this summary is that vast majority of studies use cell lines in their research with only small fraction of primary cells (Table 2). Not surprisingly, cell lines are easy to handle, they are often immortalized allowing virtually unlimited expansion of cells, cell lines are cost-effective in laboratory routine use [163]. Cell lines offer a homogeneous cell population, providing a valuable advantage by ensuring a consistent sample and reproducible outcomes [163, 164]. The utilization of cell lines has significantly transformed scientific research and advanced our knowledge in various fields from drug testing to tumor biology [163–165]. However, usage of cell lines bears certain disadvantages. Cross-contamination, misidentified cell lines, and the use of cultures at high-passage levels lead to generation of erroneous and misleading results [166–170]. For instance, analysis of various MCF-7 cell line strains revealed significant genetic heterogeneity among them [171]. When subjected to 321 anti-cancer compounds, these strains exhibited substantial variability in their responses. Notably, 75% of compounds causing marked toxicity in some strains showed complete ineffectiveness in others [171]. Another comprehensive investigation showed considerable genetic and phenotypic variability among different strains of HeLa cells [172]. These studies raise a critical question about the reproducibility of research conducted using MCF-7 and HeLa cells. It is crucial to emphasize that authenticating cell lines is essential for ensuring reproducible and reliable research [173]. Neglecting this authentication process can easily lead to erroneous outcomes, resulting in wasted time, financial resources, and compromised publication data [173]. Additionally, cell lines frequently exhibit genetic and phenotypic variations compared to their tissue of origin, whereas primary cells retain numerous crucial markers and functions observed in the in vivo environment [174, 175].

**Table 2:** Summary of mechanical cues effects on nanoparticle interactions and processing by cells

Cell type	Nanoparticle type	Nanoparticle characterization			Mechanical cue	Major outcome	References
		Diameter (nm)	Zeta potential (mV)	PDI			
BAECs	Polystyrene nanoparticles	100	NA	NA	ECM stiffness	Stiffer substrates increase total uptake nanoparticles. However, uptake per unit membrane area was found to be higher on soft substrates	[26]
Human breast cancer cell lines MCF-7 and MDA-MB-231	Pluronic F127 and TPGS mixed micelles	22.6 ± 0.7	− 0.33	0.198	ECM stiffness	MCF-7 showed stiffness-dependent uptake of micelles. MDA-MB-231 cells showed no significant differences in uptake of micelles across varying stiffnesses MCF-7 cells exhibited higher apoptosis rate at 7, 20 and 25 kPa than at 1 kPa	[25]
A549 (human alveolar type II epithelial cells) and J774A.1 (murine macrophage) cell lines	Silica particles	145 ± 4; 362 ± 8; 420 ± 13	48 ± 6.8; − 37 ± 4.5; 51 ± 2.6	0.003; 0.005; 0.005	ECM stiffness	Particle uptake is increased up to six times for A549 and two times for macrophages when cells are grown in soft substrates Smaller particles were taken more efficiently than bigger ones Expression levels of YAP protein were found to correlate with the degree of particle endocytosis	[27]
MDA-MB-231 (human breast cancer); HeLa (human cervical cancer); SK-OV-3 (human ovarian cancer); A549 (human lung cancer)	Coumarin 6-loaded poly(lactic-co-glycolic) acid micelles	101.02 ± 0.54	− 13.67 ± 1.13	0.2013	ECM stiffness	The increase in particles uptake correlated with stiffness increase Cytoskeletal tension fount to be not relevant for stiffness-related cellular uptake of particles. Higher stiffness enhanced clathrin and caveolin-1 mediated endocytosis via upregulation of protein expression levels of clathrin and caveolin-1	[176]
HeLa (human cervical cancer)	Polystyrene nanoparticles	40; 200	NA; NA	NA; NA	ECM stiffness	Soft stiffness promoted the uptake of nanoparticles from the cell ventral side. Activation of clathrin-mediated endocytosis reduced the effect of substrate stiffness on particle uptake. Myosin II is important for regulation of the effect of substrate stiffness on particle uptake	[179]
HeLa (human cervical cancer); HCT-8 (human large intestine cancer)	Polystyrene nanoparticles	100	NA	NA	ECM stiffness	Cellular uptake of nanoparticles decreases on a per-cell-area basis but increases on a per-cell basis with increasing gel stiffness	[177]
AsPC-1 (human pancreatic adenocarcinoma); MDA-MB-231 (human breast adenocarcinoma)	Polystyrene beads	800; 2400	NA; NA	NA; NA	ECM stiffness	A wave-like (“meandering”) dependence of cell uptake on the substrate stiffness	[213]
bEnd.3 (mouse brain endothelial) cells	Polystyrene nanoparticles	*Plain particles* 108.07 ± 1.12; *RGD-functionalized* 114.67 ± 1.53; *Scrambled RGD-functionalized* 110.33 ± 0.86	− 48.33 ± 0.91; − 36.17 ± 0.61; − 36.13 ± 0.84	0.005 ± 0.003; 0.07 ± 0.02; 0.04 ± 0.02	ECM stiffness	Higher stiffness promoted RGD-functionalized nanoparticles uptake by the cells	[178]
BeWo b30 cells (choriocarcinoma cell line)	PEGylated DOPC liposomes	*Plain liposomes* 48.8 ± 0.3; *CSA-conjugated liposomes* 93.7 ± 1.2	11.48 ± 4.36; − 25.5 ± 2.81	0.19 ± 0.01; 0.25 ± 0.01	Shear stress	Shear stress promoted the cell uptake of liposomes	[183]
HUVECs	Gold nanoparticles; Liposomes	*Gold nanoparticles untargeted* PEG (5 kDa)-coated; 100; *Gold nanoparticles targeted* PEG (5 kDa)-coated; UEA-1-modified; 50; *Liposomes* DSPC lipid; 126	NA; NA; NA	NA NA NA	Shear stress	Increased shear stress decreased nanoparticles uptake in endothelial cells. Decoration of the surface of particles with endothelial-cell-binding ligands partially restores uptake to nonflow levels	[187]
HEK293; HepG2 (human hepatoblastoma cell line); HUVECs; L929 (mouse fibroblast cell line); Human primary macrophages	P(MMA-co-MAA) and P(MMA-co-DMAEMA) nanoparticles	*3% PMAA* 196; *5% PMAA* 193; *8% PMAA* 207; *13% PMAA* 205; *20% PDMAEMA* 207	NA; NA; NA; NA; NA	0.061; 0.097; 0.099; 0.079; 0.101	Shear stress	Shear stress dependent increased uptake of nanoparticles was found	[184]
HeLa (human cervical cancer) and MCF-7 (human breast cancer) cell lines	DOPC lipid nanoparticles	*Unmodified particles* 143 ± 5; *PEGylated particles* 139 ± 3	34.5 ± 1.1; 19 ± 1	NA; NA	Shear stress	Shear stress increased cellular uptake of nanoparticles in HeLa cells, while it reduced their engulfment in MCF-7 cells	[188]
HUVECs	PEGDA nanoparticles	*Rods* 800 × 100 × 100 nm^3^; *Disks* Size: 325 nm diameter × 100 nm high; *Rods* Size: 400 × 100 × 100 nm^3^; *Disks* Size: 220 nm diameter × 100 nm high	57; 57; 57; 57	NA; NA; NA; NA	Shear stress	Shear stress promotes rods over discs uptake by cells	[185]
HUVECs	CdTe-QDs and SiO_2_ nanoparticles	*QD2.7* 2.7 ± 0.1; *QD4.7* 4.7 ± 0.1; *SiO*_*2*_ 50 ± 0.5	− 26 ± 7; − 47 ± 13; − 42 ± 5	NA; NA NA	Shear stress	Increased shear stress decreased nanoparticles uptake in endothelial cells	[189]
C2C12 (mouse myoblast cell line)	POPC, POPC/POEPC and POPC/POPS liposomes	~ 150	NA	0.2	Shear stress	It was found that shear stress stimulates positively charged liposomes interaction with myoblast cells	[186]
HEK	PEG-coated QDs	~ 45 nm	NA	NA	Tensile forces	Strain promoted an increase in QDs uptake	[193]
BAECs	Polystyrene nanoparticles	50; 100; 200	NA; NA; NA	NA; NA; NA	Tensile forces	Cyclic strain induced significant increase in particle uptake, which as almost linearly proportional to strain level	[214]
VSMCs	Poly(acrylic acid)-coated MNPs; Carboxymethyl-dextran-coated MNPs; Poly-L-lysine (PLL)-coated maghemite nanoparticles;	79; 200	NA; NA	NA; NA	Tensile forces	Application of cyclic strain for 4–12 h significantly reduced MNP uptake. Uptake was found to be dependent on actin polymerization	[192]
HUVECs	Silica nanoparticles	30; 70; *COOH-functionalized* 70; *NH*_*2*_*-functionalized* 70	− 20.6; − 23.4; − 29.3; − 24.6	NA; NA; NA; NA	Tensile forces	Cyclic stretch decreased endocytosis resulting in decreased uptake of nanoparticles	[208]
Xenograft of AsPC-1, rat glioscarcoma tumor cells, 9L and the human epithelial cancer in mice	FluoSpheres carboxylate-modified microsphere	40	NA	NA	Interstitial fluid pressure	Interstitial fluid pressure blocked penetration of nanoparticle into tumor tissues and concomitantly decreased cellular uptake	[194]

TPGS, D-α-tocopheryl polyethylene glycol succinate; PDI, polydispersity index; PEG, poly(ethylene glycol); DOPC, 1,2-dioleoyl-sn-glycero-3-phosphocholine; CSA, chondroitin sulphate A; HUVECs, human umbilical vein endothelial cells; BAECs, bovine aortic endothelial cells; DSPC, 1,2-distearoyl-sn-glycero-3-phosphocholine; UEA-1, Ulex europaeus agglutinin-1; HEK293, human embryonic kidney cell line; P(MMA-co-MAA), poly((methyl methacrylate)-co-(methacrylic acid)); P(MMA-co-DMAEMA), poly((methyl methacrylate)-co-(2-dimethylamino ethyl-methacrylate); PEGDA, poly(ethylene glycol) diacrylate; QDs, quantum dots; POPC, 1-palmitoyl-2-oleoyl-sn-glycero-3-phosphocholine; POPS, 1-palmitoyl-2-oleoyl-snglycero-3-phospho-L-serine (sodium salt); POEPC, 1-palmitoyl-2-oleoyl-sn-glycero-3-ethylphosphocholine; HEK, human epidermal keratinocytes; VSMCs, vascular smooth muscle cells; MNPs, nm magnetic nanoparticles; YAP, Yes associated transcriptional regulator; NA, not available. Values for nanoparticle characteristic were taken from corresponding references

**Fig. 5:**
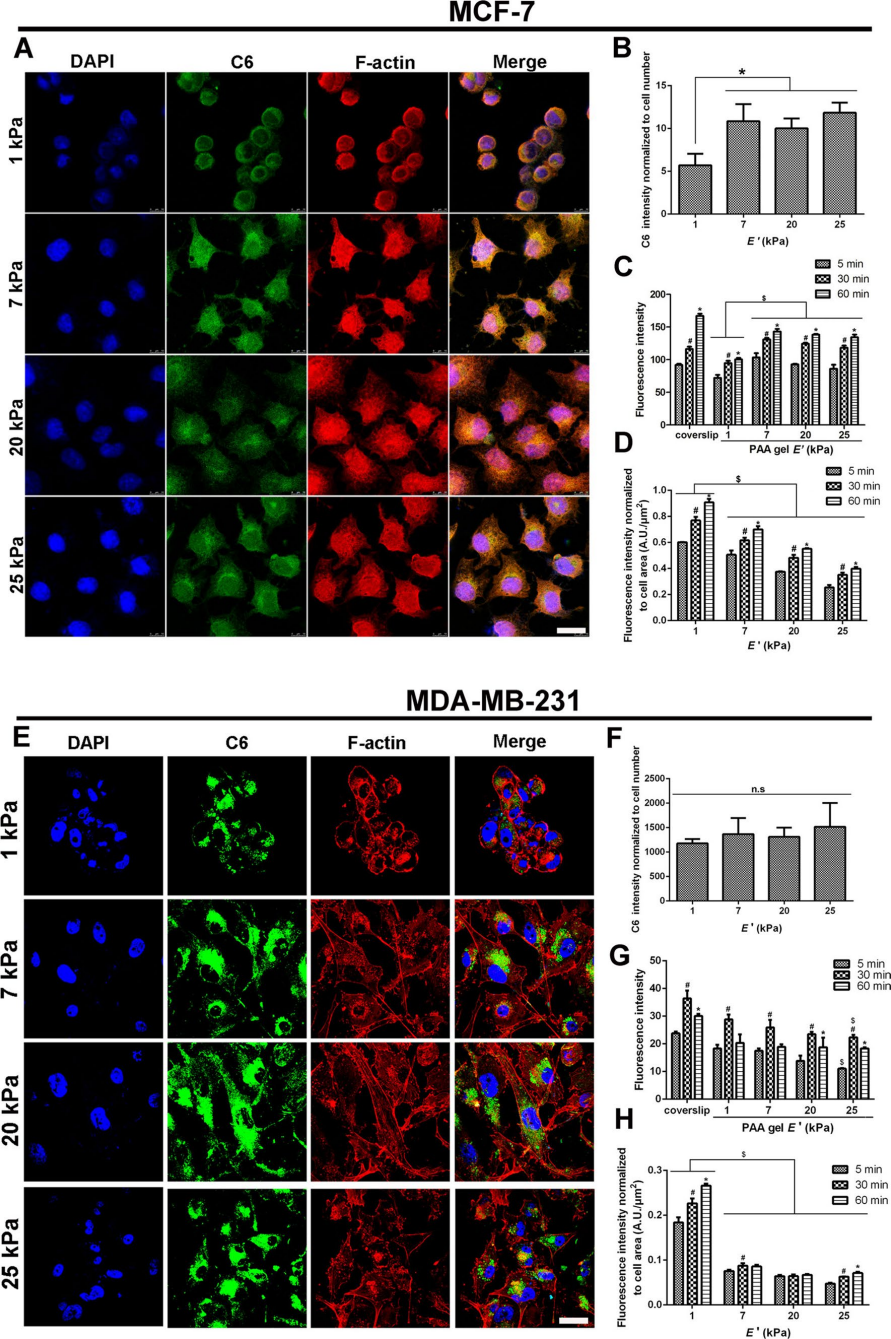
Cellular uptake of F127 MM/C6 in MCF-7 cells and MDA-MB-231 cells on PAA gel substrates. Confocal laser scanning microscopy (CLSM) images of MCF-7 cells (**A**) and MDA-MB-231 cells (**E**) on PAA substrates of varying stiffnesses. The scale bar represents 20 μm. Normalized fluorescence intensity to cell number (**B**, **F**). Fluorescence intensity was determined by a semiquantitative analysis using ImageJ software. Cell uptake efficiency over time was determined using flow cytometry (**C**, **G**). Normalized fluorescence intensity per unit area of individual cells by flow cytometry (**D**, **H**). *, Statistically different at *p* < 0.05 vs 60 min; #, statistically different at *p* < 0.05 vs 30 min; $, statistically different at *p* < 0.05 vs 1 kPa. ns, *p* > 0.05. Reprinted with permission from Wang Y, Gong T, Zhang ZR, Fu Y. Matrix stiffness differentially regulates cellular uptake behavior of nanoparticles in two breast cancer cell lines. ACS Applied Materials & Interfaces 2017;9:25,915–28 [25]. Copyright 2017 American Chemical Society

Further analysis of the literature reveals that different studies show sometimes opposite effects of same mechanical cues on nanoparticle-cell interactions (Table 2). This could be explained to some extend due to the variability of nanoparticle models and cell models utilized in the research. However, we can identify some tendencies. A number of studies have shown that the increase in particles uptake positively correlated with stiffness increase [25, 26, 176–178]. Other research has suggested that the stiffness of the substrate influences both cell adhesion and particle internalization. Softer substrates appear to enhance the levels of particle uptake [27, 179]. One can definitely say that cell type predisposes response of cells to external stiffness (Table 2). For example, MCF-7 cells were found to be sensitive to ECM stiffness in regards of uptake of nanoparticles [25]. The research has pinpointed that the efficiency of cell uptake per cell was at its minimum on soft substrates (1 kPa), and there were no noteworthy differences in nanoparticle uptake observed across substrates with stiffness ranging from 7 to 25 kPa (Fig. 5) [25]. It seems that there is a critical threshold in matrix stiffness, beyond which cellular uptake behavior ceases to respond to changes in stiffness [25]. Contrary, MDA-MB-231 cells showed no significant differences in nanoparticle uptake when cells were cultured on substrates with different stiffnesses (Fig. 5) [25]. Overall, there seems to be a consensus that smaller particles are being internalized by cells more efficiently than the bigger ones (Table 2).

Furthermore, it was found that the expression levels of YAP protein were found to correlate with the degree of particle endocytosis [27]. YAP seems to play a role in regulation of cytoskeletal dynamics, operating downstream of alterations in integrin and focal adhesion assembly, as well as in cadherin and Wnt signaling [180, 181]. Recent studies have highlighted YAP's ability to exert transcriptional control over caveolin [182]. The evidence suggests that there is a potential connection between mechanosensing of mechanical cues (e.g. stiffness and topography through integrin/adhesion machinery) and mechanical forces-driven control of endocytosis [182]. Thus, the correlation between the expression of YAP and endocytic patterns suggests that YAP's mechanosensitivity likely plays a role in regulating endocytosis and the uptake of particles through mechanical stimuli [27]. Interestingly, in the other study it was found that that cytoskeletal tension was not relevant for stiffness-related cellular uptake of particles [176]. Indeed, higher stiffness enhanced clathrin and caveolin-1 mediated endocytosis via upregulation of protein expression levels of clathrin and caveolin-1 [176].

Similarly to the effects of ECM stiffness, shear stress showed to be either promoting factor that stimulates nanoparticles uptake [183–186] or strongly inhibited endocytosis of nanoparticles (Table 2) [187–189]. Once again, cellular response is dependent on cell type utilized in the study (Table 2). For instance, it was found that shear stress enhances cellular uptake of nanoparticles in HeLa cells, contrary it reduced their internalization in MCF-7 cells [188]. Further, it was found that shear stress effect on particle uptake is dependent on shape and size of the particles [185]. Negatively charged rod-shaped PEG nanoparticles with varying aspect ratios were utilized to assess their uptake by HUVECs in flow conditions at different incubation durations (1, 12, and 24 h) [185]. Consistently, the uptake of larger particles surpassed that of smaller ones under flow, as opposed to static culture. Conversely, smaller particles exhibited higher internalization in static conditions compared to flow conditions [185]. Form the other hand, it was shown that cell viability is significantly higher when assessing gold nanoparticle under flow conditions as opposed to static culture [190]. This effect was found to be not dependent on nanoparticle size or concentration [190]. Interestingly, the other study revealed that the cell viability is promoted under flow conditions, compared to static culture [191]. Moreover, particles with low density exhibited reduced uptake under flow conditions in comparison to particles with high density [191].

Another important parameter that affects nanoparticle-cell interactions under flow conditions is surface charge. For example, it was shown that positively charged liposomes lead to increased cellular interaction in the presence of shear stress, as opposed to lipids with negative charges or those that are zwitterionic [186]. Additionally, the study explored the therapeutic impact on cell viability following treatment with positively charged liposomes containing a small cytotoxic molecule under both static and dynamic conditions [186]. The results indicated elevated therapeutic response manifested by increased cell mortality, particularly in dynamic conditions [186]. This underscores the connection between the enhanced cellular association of positively charged carriers and a more effective therapeutic outcome in the presence of shear stress [186].

The analysis of effects of tensile forces on nanoparticle-cell interactions reveals that tensile forces (similarly with ECM stiffness of shear stress) may either promote or inhibit the uptake of nanoparticles by cells (Table 2). For example, the application of cyclic strain resulted in a significant reduction of nanoparticle uptake in vascular smooth muscle cells (VSMCs) [192]. This reduction was associated with the attenuation of microvilli and a decrease in vesicle size per cell, although not in the number of vesicles per cell [192]. Interestingly, both cyclic strain and fibronectin coating of the culture plate led to a decrease in internalized nanoparticles, which were co-localized with vinculin [192]. Additionally, it was found that nanoparticle uptake was dependent on actin polymerization but not on myosin II [192]. Contrary, the other study showed that cyclic tensile strain enhances interactions between human epidermal keratinocytes and quantum dot nanoparticles resulting into elevated uptake due to increased cell membrane permeability [193].

It is worth noting that it is difficult to analyze the effect of hydrostatic pressure on nanoparticle-cell interactions due to the limited number of studies (Table 2). Lack of studies can be explained by the complexity of the experimental model that often requires in vivo conditions (Table 2). Indeed, increased interstitial fluid pressure was shown to block penetration of nanoparticle into tumor tissues and concomitantly decreased cellular uptake [194].

It becomes evident that the cellular mechanical microenvironment and physical factors play a crucial role in regulating cell homeostasis and function [17–20]. Despite the various barriers that nanomedicine must navigate to reach its target site, the therapeutic effectiveness is seemed to be predominantly determined by the nanoparticles' efficiency in entering cells [195]. This process encompasses both the uptake of nanoparticles by cells and their distribution within blood vessels at the target site [195]. Therefore, understanding how given mechanical factor bias nanoparticle-cell interactions could be beneficial for the development of better controlled and more effective nanomedicine-based therapeutic treatment strategies.

## Mechanical cues alter the endocytosis pathways of nanoparticles

**Fig. 6:**
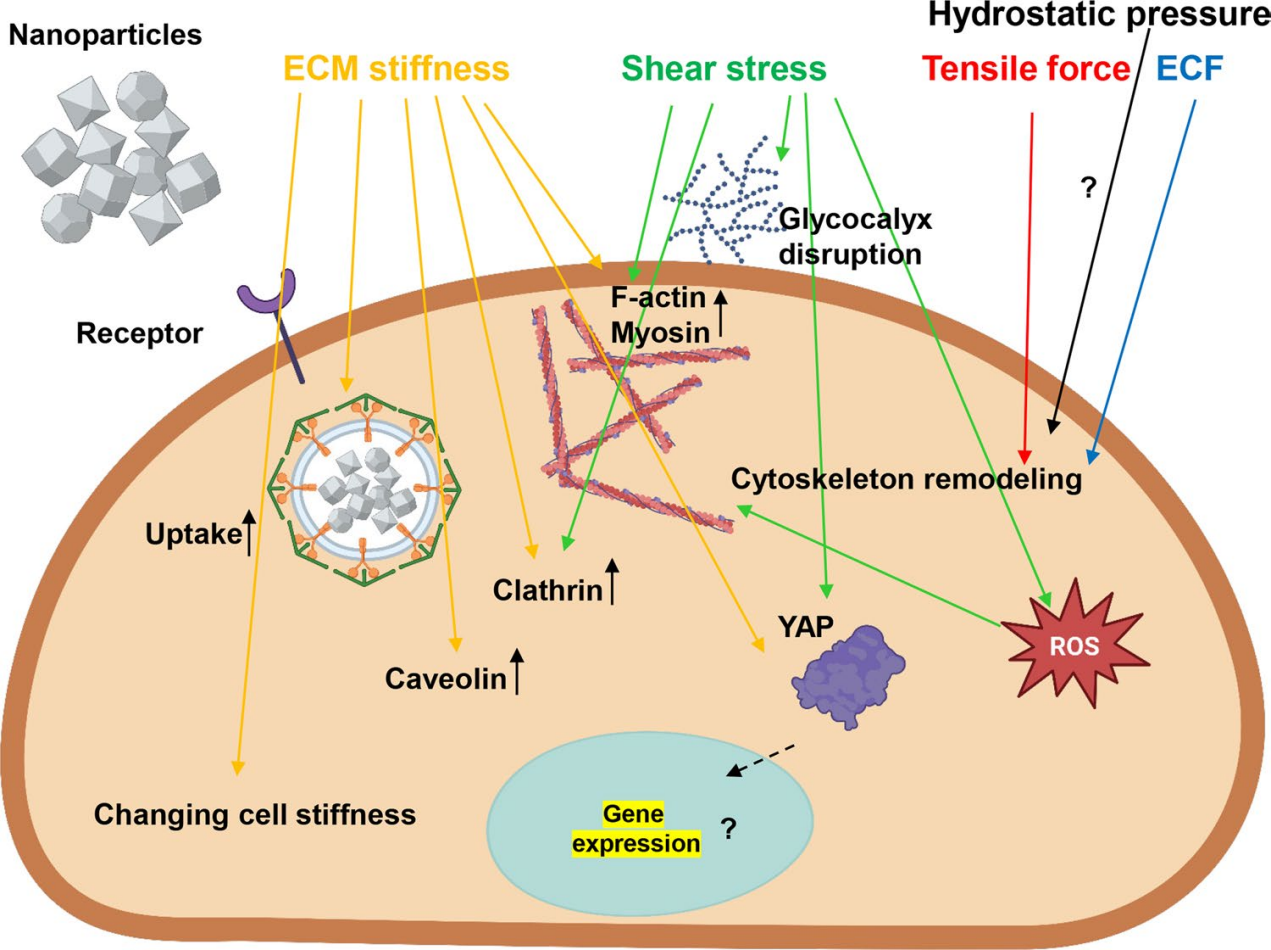
Schematic representation of molecular mechanisms how different mechanical cues (tensile force, extracellular fluid viscosity, hydrostatic pressure, and shear stress) regulate nanoparticle uptake by cells. Red arrows indicate tensile forces, green arrows indicate shear stress, blue arrows indicate impact of extracellular fluid viscosity, and black arrows refer to hydrostatic pressure, yellow arrows indicate effect of ECM stiffness. ECM, extracellular matrix; ECF, extracellular fluid viscosity; YAP, yes-associated protein; ROS, reactive oxygen species. Created with BioRender.com

It is worth noting here, that the effects of mechanical stimuli on nanoparticle uptake should be reflected by regulating the mechanical properties or stress state of the cell or by altering the endocytosis pathways (Fig. 6). Thus, we analyzed further what potential molecular cellular mechanisms could be responsible for the impact of mechanical stimuli on the total uptake level of nanoparticles (Table 2). It becomes evident that mechanical stimuli (e.g. ECM stiffness and architecture) may greatly affect the intracellular mechanical state of cells (e.g. changing mechanical properties of cells and/or influencing the stress distribution within the cell) [19, 196, 197]. These changes in cell state may ultimately translate into the regulation of nanoparticle uptake. However, studies analyzing how mechanical stimuli driven changes in the intracellular mechanical state affects nanoparticle uptake are currently very limited (Table 2). The intracellular mechanical state is tightly connected with cytoskeletal remodeling [19]. Non-surprisingly, it was found that ECM stiffness regulates cellular uptake of nanoparticles via actin cytoskeleton remodeling [26]. In soft ECM, round cells lacked stress fibers, only discrete bright spots were observed. Conversely, cells on stiff ECM displayed significantly more aligned stress fibers compared to those on intermediate ECM [26]. In fact, it was found that the cell spreading positively correlates with F-actin fiber remodeling [26].

However, emerging evidence suggests that mechanical stimuli may regulate nanoparticle uptake via altering the endocytosis pathways (Fig. 6 and Table 2). For example, it was found that stiffness may regulate nanoparticle uptake via modulation of clathrin expression and thereby affecting endocytosis rate [25]. The study demonstrated that the stiffness of the matrix affects both actin polymerization and clathrin levels. The findings indicate that variations in actin polymerization and clathrin expression across different substrate stiffnesses collectively play a role in governing uptake behavior [25]. In general, recent findings indicate that the regulation of particle uptake is mainly driven by alterations in clathrin and caveolae-mediated endocytosis [176]. From the other hand, YAP expression is being recognized as main mechanistic regulator of the interplay between mechanoregulatory networks and endocytosis [198]. YAP seems to govern cytoskeletal dynamics, operating downstream of alterations in integrin and focal adhesion assembly, as well as in cadherin and Wnt signaling pathways [181]. In fact, it was found that the expression levels of YAP protein correlate with the degree of particle endocytosis [27]. YAP serves as a key signaling component within the Hippo pathway and is known to be sensitive to alterations in the physical microenvironment [181]. Cellular structures like focal adhesions, caveolae, and clathrin-coated pits play crucial roles in transmitting mechanical cues to the cell, responding to both cellular and membrane stresses [181]. Changes in substrate stiffness and topography lead to observable shifts in focal adhesion size and morphology, influencing mechanobiological signaling pathways [199, 200]. The fluctuations in cytoskeletal stress associated with focal adhesion maturation can be effectively sensed by YAP. Further, YAP may modulate cytoskeletal elements assembly and turnover through feedback mechanisms [201]. When YAP levels are diminished, cells exhibit reduced migratory persistence and heightened cellular stress, accompanied by the maturation and stabilization of larger focal adhesions [201]. Conversely, overexpression of YAP in cells cultured on soft substrates may account for the observed decrease in focal adhesions and a concurrent reduction in cellular stress, potentially promoting endocytosis [177].

Overall, it seems that regulation of nanoparticle uptake by substrate stiffness via modulating the endocytosis through cytoskeletal and motor proteins is a general mechanism found in different cell lines and with various nanoparticles (Fig. 6 and Table 2). Hence, the activation of clathrin-mediated endocytosis has been observed to mitigate the influence of substrate stiffness on particle uptake [179]. Additionally, Myosin II plays a crucial role in regulating the impact of substrate stiffness on particle uptake. This suggests that both clathrin-mediated endocytosis and Myosin II activity are key factors in modulating how substrate stiffness influences the uptake of particles [179].

Current studies on the effect of shear stress on nanoparticle uptake suggest that uptake may be influenced by shear stress through alterations in cell morphology, the cytoskeleton, permeability, membrane fluidity and the expression of important markers and surface proteins [195]. Similarly to studies on the effect of substrate stiffness, shear stress regulates nanoparticle uptake via cytoskeletal remodeling (Fig. 6 and Table 2). It's important to highlight that shear stress might indirectly influence the uptake of cell-derived particles, such as red blood cell extracellular vesicles, by altering the levels of intracellular reactive oxygen species (ROS) [195]. It was found that oxidative stress induced by shear stress serves as a regulator for the uptake of red blood cell extracellular vesicles (RBCEVs) by endothelial cells (ECs) [202]. Shear stress was found to elevate the levels of superoxide dismutase (SOD) protein while reducing the levels of malondialdehyde (MDA) protein, indicating a rapid induction of oxidative stress. It has been established that shear stress induces oxidative stress in ECs, consequently enhancing the uptake of RBCEVs [202].

Further, shear stress may regulate nanoparticle uptake via glycocalyx modulation [203]. The endothelial glycocalyx plays a crucial role in EC response to shear stress. Recent study revealed that application of 12 dyne/cm^2^ shear stress may mechanistically damage the endothelial glycocalyx. Consequently, the uptake of gold nanoparticles by ECs increased alongside endothelial glycocalyx damage [203]. Additionally, when subjected to shear stress, the lipid order of endothelial cell membranes undergoes a transition from a state of liquid order to one of liquid disorder, accompanied by an increase in membrane fluidity [204]. This alteration in membrane properties has significant implications for nanoparticle uptake by cells, as the degree of membrane fluidity correlates with an increase in nanoparticle uptake [205].

Similarly to substrate stiffness shear stress may regulate nanoparticle uptake via modulating clathrin expression (Fig. 6 and Table 2). Shear stress was found to increase progressively the uptake of polystyrene by endothelial cells [206]. Analysis of clathrin expression levels under different shear stress conditions suggested that the augmented uptake was attributed to a shear-dependent elevation in clathrin levels [206]. Subsequently, it was shown that shear stress upregulated clathrin expression in HK-2 cells, leading to increased nanoparticles uptake [207].

It's important to highlight that research examining the impact of tensile forces and interstitial fluid pressure on nanoparticle uptake is relatively limited (Table 2). The limited availability of experimental data hinders the ability to draw sound conclusions regarding the mechanisms through which tensile forces and interstitial fluid pressure may influence nanoparticle uptake. However, insights gleaned from existing literature suggest that alterations in actin cytoskeleton assembly could potentially serve as a primary mechanism underlying the effects of tensile forces on nanoparticle uptake [192, 208]. Further investigation in this area is needed to elucidate the precise relationship between tensile forces or interstitial fluid pressure and nanoparticle uptake.

## Outlook and perspectives

Typically, nanomaterials undergo preliminary testing for biomedical applications using standard 2D cell culture techniques [6, 11, 13, 209]. While cell culture is a convenient method for assessing the toxicity and efficacy of nanomaterials, its utility is constrained by certain limitations. One significant challenge is the potential for particle aggregation and sedimentation, which may not accurately reflect the behavior of nanomaterials in more complex biological environments [6, 11, 13, 209]. Furthermore, cell culture conditions may not entirely mimic the native conditions found in animal models or the human body, raising concerns about the translation of findings from cell-based assays to in vivo conditions [6, 11, 13, 209]. Therefore, while standard cell cultures serve as a valuable initial testing basis, subsequent studies using more representative model systems are crucial for a comprehensive understanding of nanomaterial behavior in biological contexts. Importantly, one can see from our analysis presented here that physical factors (e.g. hydrostatic pressure, tensile forces, stiffness of ECM, extracellular fluid viscosity) play an important role in the regulation of nanoparticle-cell interactions. In this review, we have compiled insights into the interplay between those mechanical cues and nanoparticle-cell interactions (Table 2). The uptake of nanomaterials under the influence of physical factors is affected by a myriad of factors, some related to the nanomaterials themselves, while others are cell specific. Critical nanomaterial characteristics such as size, shape, surface charge, surface ligands, and particle elasticity play pivotal roles in cellular uptake under the influence of physical factors [209]. However, it is essential to acknowledge that these factors exert diverse effects on the interaction between nanomaterials and cells, and their impact can vary depending on the specific cell type. The type of cell, as well as the tissue origin, introduces additional complexities. Consequently, there is no overarching rule governing how nanomaterials will universally interact with cells. The detailed interplay between nanomaterial properties and cellular responses underscores the need for a context-specific understanding, emphasizing the importance of tailored investigations for both cancer and normal cell scenarios in order to unravel the intricacies of nanomaterial-cell interactions under the influence of physical factors.

Further, we highlighted in this review that the majority of studies addressing the influence of physical factors on nanomaterial-cell interactions are performed utilizing standard cell lines as cellular models. Only a limited number of studies utilize primary cell cultures to explore how mechanical cues bias nanoparticle-cell interactions (Table 2). Moreover, a significant proportion of research still relies on potentially problematic cell lines, such as HeLa and MCF-7 (refer to Table 2), posing a potential impact on the reproducibility of their findings.

Another challenge that we can spot is relatively limited use of 3D cell culture models to decipher effects of physical factors on nanoparticle-cell uptake and processing by cells. It is understandable that 2D systems provide more controllable and easier to handle environments for cell analysis. However, 3D culture systems may provide a platform to study effects of several physical factors simultaneously [24, 130–132, 210].

In conclusion, it is important to underscore that current research directions realize the substantial impact of physical factors on nanoparticle-cell interactions. Studies take into account the complexity of in vivo conditions and try to mimic them in various experimental systems. Anticipating a broader spectrum of applications for nanoparticles that can transition into clinical utility is a reasonable expectation. However, it is equally crucial to comprehend the obstacles and challenges associated with therapeutic nanotechnology to ensure its clinical success. Therefore, we hope that current review might serve as a catalyst for novel ideas and innovative approaches in the design of safe and effective nanomedicines tailored for specific applications. Comprehensive understanding of how mechanical cues bias nanoparticle-cell interactions will bring valuable fundamental knowledge, that in future will enable formulation of a strategic roadmap for advancing the nanobio-field toward novel successful clinical implementation.

## Data Availability

Not applicable.

## References

[1] Mitchell MJ, Billingsley MM, Haley RM, Wechsler ME, Peppas NA, Langer R (2021). Engineering precision nanoparticles for drug delivery. Nat Rev Drug Discov.

[2] Poon W, Kingston BR, Ouyang B, Ngo W, Chan WCW (2020). A framework for designing delivery systems. Nat Nanotechnol.

[3] Shi JJ, Kantoff PW, Wooster R, Farokhzad OC (2017). Cancer nanomedicine: progress, challenges and opportunities. Nat Rev Cancer.

[4] Placci M, Giannotti MI, Muro S (2023). Polymer-based drug delivery systems under investigation for enzyme replacement and other therapies of lysosomal storage disorders. Adv Drug Deliv Rev.

[5] Blanco E, Shen H, Ferrari M (2015). Principles of nanoparticle design for overcoming biological barriers to drug delivery. Nat Biotechnol.

[6] Frtus A, Smolkova B, Uzhytchak M, Lunova M, Jirsa M, Henry SJW (2022). The interactions between DNA nanostructures and cells: a critical overview from a cell biology perspective. Acta Biomater.

[7] Stephanopoulos N (2020). Hybrid nanostructures from the self-assembly of proteins and DNA. Chem.

[8] Henry SJW, Stephanopoulos N (2021). Functionalizing DNA nanostructures for therapeutic applications. Wiley Interdiscip Rev-Nanomed Nanobiotechnol.

[9] Szebeni J, Storm G, Ljubimova JY, Castells M, Phillips EJ, Turjeman K (2022). Applying lessons learned from nanomedicines to understand rare hypersensitivity reactions to mRNA-based SARS-CoV-2 vaccines. Nat Nanotechnol.

[10] Yang W, Wang L, Mettenbrink EM, DeAngelis PL, Wilhelm S (2021). Nanoparticle toxicology. Annu Rev Pharmacol Toxicol.

[11] Frtús A, Smolková B, Uzhytchak M, Lunova M, Jirsa M, Kubinová S (2020). Analyzing the mechanisms of iron oxide nanoparticles interactions with cells: a road from failure to success in clinical applications. J Control Release.

[12] Sharma S, Parveen R, Chatterji BP (2021). Toxicology of nanoparticles in drug delivery. Curr Pathobiol Rep.

[13] Uzhytchak M, Smolková B, Lunova M, Frtús A, Jirsa M, Dejneka A (2023). Lysosomal nanotoxicity: impact of nanomedicines on lysosomal function. Adv Drug Deliv Rev.

[14] Lunova M, Smolková B, Lynnyk A, Uzhytchak M, Jirsa M, Kubinová S (2019). Targeting the mTOR signaling pathway utilizing nanoparticles: A critical overview. Cancers.

[15] Mitragotri S, Lammers T, Bae YH, Schwendeman S, De Smedt S, Leroux JC (2017). Drug delivery research for the future: expanding the nano horizons and beyond. J Control Release.

[16] Hua S, de Matos MBC, Metselaar JM, Storm G (2018). Current trends and challenges in the clinical translation of nanoparticulate nanomedicines: pathways for translational development and commercialization. Front Pharmacol.

[17] Saraswathibhatla A, Indana D, Chaudhuri O (2023). Cell-extracellular matrix mechanotransduction in 3D. Nat Rev Mol Cell Biol.

[18] Uhler C, Shivashankar GV (2017). Regulation of genome organization and gene expression by nuclear mechanotransduction. Nat Rev Mol Cell Biol.

[19] Janmey PA, Fletcher DA, Reinhart-King CA (2020). Stiffness sensing by cells. Physiol Rev.

[20] Yamada KM, Doyle AD, Lu JY (2022). Cell-3D matrix interactions: recent advances and opportunities. Trends Cell Biol.

[21] Romani P, Valcarcel-Jimenez L, Frezza C, Dupont S (2021). Crosstalk between mechanotransduction and metabolism. Nat Rev Mol Cell Biol.

[22] Ladoux B, Mège RM (2017). Mechanobiology of collective cell behaviours. Nat Rev Mol Cell Biol.

[23] Du HX, Bartleson JM, Butenko S, Alonso V, Liu WF, Winer DA (2023). Tuning immunity through tissue mechanotransduction. Nat Rev Immunol.

[24] Di XP, Gao XS, Peng L, Ai JZ, Jin X, Qi SQ (2023). Cellular mechanotransduction in health and diseases: from molecular mechanism to therapeutic targets. Signal Transduct Target Ther.

[25] Wang Y, Gong T, Zhang ZR, Fu Y (2017). Matrix stiffness differentially regulates cellular uptake behavior of nanoparticles in two breast cancer cell lines. ACS Appl Mater Interfaces.

[26] Huang CJ, Butler PJ, Tong S, Muddana HS, Bao G, Zhang SL (2013). Substrate stiffness regulates cellular uptake of nanoparticles. Nano Lett.

[27] Lee AR, de Almeida MS, Milinkovic D, Septiadi D, Taladriz-Blanco P, Loussert-Fonta C (2022). Substrate stiffness reduces particle uptake by epithelial cells and macrophages in a size-dependent manner through mechanoregulation. Nanoscale.

[28] Cox TR (2021). The matrix in cancer. Nat Rev Cancer.

[29] Hanahan D (2022). Hallmarks of cancer: new dimensions. Cancer Discov.

[30] Nia HDT, Munn LL, Jain RK (2020). Physical traits of cancer. Science.

[31] Paul CD, Mistriotis P, Konstantopoulos K (2017). Cancer cell motility: lessons from migration in confined spaces. Nat Rev Cancer.

[32] Petridou NI, Spiró Z, Heisenberg CP (2017). Multiscale force sensing in development. Nat Cell Biol.

[33] Humphrey JD, Dufresne ER, Schwartz MA (2014). Mechanotransduction and extracellular matrix homeostasis. Nat Rev Mol Cell Biol.

[34] Iskratsch T, Wolfenson H, Sheetz MP (2014). Appreciating force and shape - the rise of mechanotransduction in cell biology. Nat Rev Mol Cell Biol.

[35] Discher DE, Smith L, Cho S, Colasurdo M, García AJ, Safran S (2017). Matrix mechanosensing: from scaling concepts in 'omics data to mechanisms in the nucleus, regeneration, and cancer. Ann Rev Biophys.

[36] Evers TMJ, Holt LJ, Alberti S, Mashaghi A (2021). Reciprocal regulation of cellular mechanics and metabolism. Nat Metab.

[37] Vining KH, Mooney DJ (2017). Mechanical forces direct stem cell behaviour in development and regeneration. Nat Rev Mol Cell Biol.

[38] Fernandez-Sanchez ME, Brunet T, Röper JC, Farge E (2015). Mechanotransduction's impact on animal development, evolution, and tumorigenesis. Annu Rev Cell Dev Biol.

[39] Mascharak S, Talbott HE, Januszyk M, Griffin M, Chen K, Davitt MF (2022). Multi-omic analysis reveals divergent molecular events in scarring and regenerative wound healing. Cell Stem Cell.

[40] Marinval N, Chew SY (2021). Mechanotransduction assays for neural regeneration strategies: a focus on glial cells. APL Bioeng.

[41] Long Y, Niu YD, Liang KN, Du YN (2022). Mechanical communication in fibrosis progression. Trends Cell Biol.

[42] Cooper J, Giancotti FG (2019). Integrin signaling in cancer: Mechanotransduction, stemness, epithelial plasticity, and therapeutic resistance. Cancer Cell.

[43] Zhu PF, Lu HR, Wang MX, Chen K, Chen ZL, Yang L (2023). Targeted mechanical forces enhance the effects of tumor immunotherapy by regulating immune cells in the tumor microenvironment. Cancer Biol Med.

[44] Li N, Zhang XY, Zhou J, Li W, Shu XY, Wu Y (2022). Multiscale biomechanics and mechanotransduction from liver fibrosis to cancer. Adv Drug Deliv Rev.

[45] Heldin CH, Rubin K, Pietras K, Östman A (2004). High interstitial fluid pressure - an obstacle in cancer therapy. Nat Rev Cancer.

[46] Henstock JR, Rotherham M, Rose JB, El Haj AJ (2013). Cyclic hydrostatic pressure stimulates enhanced bone development in the foetal chick femur. Bone.

[47] Nessler KHL, Henstock JR, El Haj AJ, Waters SL, Whiteley JP, Osborne JM (2016). The influence of hydrostatic pressure on tissue engineered bone development. J Theor Biol.

[48] Fukuchi M, Oyama K, Mizuno H, Miyagawa A, Koumoto K, Fukuhara G (2021). Hydrostatic pressure-regulated cellular calcium responses. Langmuir.

[49] Sugimoto A, Miyazaki A, Kawarabayashi K, Shono M, Akazawa Y, Hasegawa T (2017). Piezo type mechanosensitive ion channel component 1 functions as a regulator of the cell fate determination of mesenchymal stem cells. Sci Rep.

[50] Li X, Xue YM, Guo HM, Deng CY, Peng DW, Yang H (2020). High hydrostatic pressure induces atrial electrical remodeling through upregulation of inflammatory cytokines. Life Sci.

[51] Huang ZS, Khalifa MO, Gu WL, Li TS (2022). Hydrostatic pressure induces profibrotic properties in hepatic stellate cells via the RhoA/ROCK signaling pathway. FEBS Open Bio.

[52] Huang ZS, Khalifa MO, Li PL, Huang Y, Gu WL, Li TS (2022). Angiotensin receptor blocker alleviates liver fibrosis by altering the mechanotransduction properties of hepatic stellate cells. Am J Physiol-Gastroint Liver Physiol.

[53] Salker MS, Steel JH, Hosseinzadeh Z, Nautiyal J, Webster Z, Singh Y (2016). Activation of SGK1 in endometrial epithelial cells in response to PI3K/AKT inhibition impairs embryo implantation. Cell Physiol Biochem.

[54] Mihic A, Li J, Miyagi Y, Gagliardi M, Li SH, Zu J (2014). The effect of cyclic stretch on maturation and 3D tissue formation of human embryonic stem cell-derived cardiomyocytes. Biomaterials.

[55] Zhou L, Wei X, Liu Q, Cai X, Liao BH, Yang J (2017). Hydrostatic pressure and muscarinic receptors are involved in the release of inflammatory cytokines in human bladder smooth muscle cells. Neurourol Urodyn.

[56] Gao XS, Wei TQ, Chen JX, Ai JZ, Jin T, Cheng L (2018). Cyclic hydrostatic pressure promotes uroplakin expression in human urothelial cells through activation of ERK1/2 signaling. Biochem Biophys Res Commun.

[57] Abidin NAZ, Timofeeva M, Szydzik C, Akbaridoust F, Lav C, Marusic I (2023). A microfluidic method to investigate platelet mechanotransduction under extensional strain. Res Pract Thromb Haemost.

[58] Feinberg MW, Moore KJ (2016). MicroRNA regulation of atherosclerosis. Circ Res.

[59] Yamawaki H, Pan S, Lee RT, Berk BC (2005). Fluid shear stress inhibits vascular inflammation by decreasing thioredoxin-interacting protein in endothelial cells. J Clin Invest.

[60] Chiu JJ, Chen CN, Lee PL, Yang CT, Chuang HS, Chien S (2003). Analysis of the effect of disturbed flow on monocytic adhesion to endothelial cells. J Biomech.

[61] Gray KM, Stroka KM (2017). Vascular endothelial cell mechanosensing: New insights gained from biomimetic microfluidic models. Semin Cell Dev Biol.

[62] Tarbell JM, Shi ZD, Dunn J, Jo H (2014). Fluid mechanics, arterial disease, and gene expression. Annu Rev Fluid Mech.

[63] Papaioannou TG, Stefanadis C (2005). Vascular wall shear stress: basic principles and methods. Hell J Cardiol.

[64] Caro CG (2009). Discovery of the role of wall shear in atherosclerosis. Arterioscler Thromb Vasc Biol.

[65] Fujiwara K (2006). Platelet endothelial cell adhesion molecule-1 and mechanotransduction in vascular endothelial cells. J Intern Med.

[66] Liu ZQ, Ruter DL, Quigley K, Tanke NT, Jiang YC, Bautch VL (2021). Single-cell RNA sequencing reveals endothelial cell transcriptome heterogeneity under homeostatic laminar flow. Arterioscler Thromb Vasc Biol.

[67] Shinge SAU, Zhang D, Achu Muluh T, Nie Y, Yu F (2021). Mechanosensitive Piezo1 channel evoked-mechanical signals in atherosclerosis. J Inflamm Res.

[68] Paudel R, Fusi L, Schmidt M (2021). The MEK5/ERK5 pathway in health and disease. Int J Mol Sci.

[69] Givens C, Tzima E (2016). Endothelial mechanosignaling: Does one sensor fit all?. Antioxid Redox Signal.

[70] Poelmann RE, Gittenberger-de Groot AC (2018). Hemodynamics in cardiac development. J Cardiovasc Dev Dis.

[71] Jetta D, Gottlieb PA, Verma D, Sachs F, Hua SZ (2019). Shear stress-induced nuclear shrinkage through activation of Piezo1 channels in epithelial cells. J Cell Sci.

[72] Tzima E, Irani-Tehrani M, Kiosses WB, Dejana E, Schultz DA, Engelhardt B (2005). A mechanosensory complex that mediates the endothelial cell response to fluid shear stress. Nature.

[73] Conway DE, Breckenridge MT, Hinde E, Gratton E, Chen CS, Schwartz MA (2013). Fluid shear stress on endothelial cells modulates mechanical tension across VE-cadherin and PECAM-1. Curr Biol.

[74] Shiu YT, Li S, Marganski WA, Usami S, Schwartz MA, Wang YL (2004). Rho mediates the shear-enhancement of endothelial cell migration and traction force generation. Biophys J.

[75] Tzima E (2006). Role of small GTPases in endothelial cytoskeletal dynamics and the shear stress response. Circ Res.

[76] Obi S, Masuda H, Shizuno T, Sato A, Yamamoto K, Ando J (2012). Fluid shear stress induces differentiation of circulating phenotype endothelial progenitor cells. Am J Physiol-Cell Physiol.

[77] Tijore A, Yao MX, Wang YH, Hariharan A, Nematbakhsh Y, Doss BL (2021). Selective killing of transformed cells by mechanical stretch. Biomaterials.

[78] Opplert J, Babault N (2018). Acute effects of dynamic stretching on muscle flexibility and performance: An analysis of the current literature. Sports Med.

[79] Camasao DB, Mantovani D (2021). The mechanical characterization of blood vessels and their substitutes in the continuous quest for physiological-relevant performances. A Crit Rev Mater Today Bio.

[80] Rysä J, Tokola H, Ruskoaho H (2018). Mechanical stretch induced transcriptomic profiles in cardiac myocytes. Sci Rep.

[81] Albinsson S, Bhattachariya A, Hellstrand P (2014). Stretch-dependent smooth muscle differentiation in the portal vein-role of actin polymerization, calcium signaling, and microRNAs. Microcirculation.

[82] Gao X, Wei T, Liao B, Ai J, Zhou L, Gong L (2018). Physiological stretch induced proliferation of human urothelial cells via integrin alpha6-FAK signaling pathway. Neurourol Urodyn.

[83] Loverde JR, Tolentino RE, Soteropoulos P, Pfister BJ (2020). Biomechanical forces regulate gene transcription during stretch-mediated growth of mammalian neurons. Front Neurosci.

[84] Purohit PK, Smith DH (2016). A model for stretch growth of neurons. J Biomech.

[85] Liu B, Qu MJ, Qin KR, Li H, Li ZK, Shen BR (2008). Role of cyclic strain frequency in regulating the alignment of vascular smooth muscle cells in vitro. Biophys J.

[86] Chen QH, Li W, Quan ZW, Sumpio BE (2003). Modulation of vascular smooth muscle cell alignment by cyclic strain is dependent on reactive oxygen species and P38 mitogen-activated protein kinase. J Vasc Surg.

[87] Tock J, Van Putten V, Stenmark KR, Nemenoff RA (2003). Induction of SM-α-actin expression by mechanical strain in adult vascular smooth muscle cells is mediated through activation of JNK and p38 MAP kinase. Biochem Biophys Res Commun.

[88] Numaguchi K, Eguchi S, Yamakawa T, Motley ED, Inagami T (1999). Mechanotransduction of rat aortic vascular smooth muscle cells requires RhoA and intact actin filaments. Circ Res.

[89] Theocharis AD, Skandalis SS, Gialeli C, Karamanos NK (2016). Extracellular matrix structure. Adv Drug Deliv Rev.

[90] Marchioni A, Tonelli R, Cerri S, Castaniere I, Andrisani D, Gozzi F (2021). Pulmonary stretch and lung mechanotransduction: implications for progression in the fibrotic lung. Int J Mol Sci.

[91] Karsdal MA, Nielsen SH, Leeming DJ, Langholm LL, Nielsen MJ, Manon-Jensen T (2017). The good and the bad collagens of fibrosis - Their role in signaling and organ function. Adv Drug Deliv Rev.

[92] López B, Ravassa S, Moreno MU, San José G, Beaumont J, González A (2021). Diffuse myocardial fibrosis: mechanisms, diagnosis and therapeutic approaches. Nat Rev Cardiol.

[93] López B, González A, Querejeta R, Larman M, Rábago G, Díez J (2014). Association of cardiotrophin-1 with myocardial fibrosis in hypertensive patients with heart failure. Hypertension.

[94] Echegaray K, Andreu I, Lazkano A, Villanueva I, Sáenz A, Elizalde MR (2017). Role of myocardial collagen in severe aortic stenosis with preserved ejection fraction and symptoms of heart failure. Rev Esp Cardiol.

[95] Kisseleva T, Brenner D (2021). Molecular and cellular mechanisms of liver fibrosis and its regression. Nat Rev Gastroenterol Hepatol.

[96] Bataller R, Brenner DA (2005). Liver fibrosis. J Clin Invest.

[97] Cubero FJ, Urtasun R, Nieto N (2009). Alcohol and liver fibrosis. Semin Liver Dis.

[98] Arriazu E, de Galarreta MR, Cubero FJ, Varela-Rey M, de Obanos MPP, Leung TM (2014). Extracellular matrix and liver disease. Antioxid Redox Signal.

[99] Cai JY, Hu M, Chen ZY, Ling Z (2021). The roles and mechanisms of hypoxia in liver fibrosis. J Transl Med.

[100] Chuliá-Peris L, Carreres-Rey C, Gabasa M, Alcaraz J, Carretero J, Pereda J (2022). Matrix metalloproteinases and their inhibitors in pulmonary fibrosis: EMMPRIN/CD147 comes into play. Int J Mol Sci.

[101] Liu F, Mih JD, Shea BS, Kho AT, Sharif AS, Tager AM (2010). Feedback amplification of fibrosis through matrix stiffening and COX-2 suppression. J Cell Biol.

[102] Masuzaki R, Tateishi R, Yoshida H, Goto E, Sato T, Ohki T (2009). Prospective risk assessment for hepatocellular carcinoma development in patients with chronic hepatitis C by transient elastography. Hepatology.

[103] Castera L (2009). Liver stiffness and hepatocellular carcinoma: liaisons dangereuses?. Hepatology.

[104] Choong KL, Wong YH, Yeong CH, Gnanasuntharam GK, Goh KL, Yoong BK (2017). Elasticity characterization of liver cancers using shear wave ultrasound elastography: comparison between hepatocellular carcinoma and liver metastasis. J Diagn Med Sonog.

[105] Masuzaki R, Tateishi R, Yoshida H, Sato T, Ohki T, Goto T (2007). Assessing liver tumor stiffness by transient elastography. Hepatol Int.

[106] Xia TT, Zhao RZ, Liu WQ, Huang QP, Chen PX, Waju YN (2018). Effect of substrate stiffness on hepatocyte migration and cellular Young's modulus. J Cell Physiol.

[107] Takeda T, Yasuda T, Nakayama Y, Nakaya M, Kimura M, Yamashita M (2006). Usefulness of noninvasive transient elastography for assessment of liver fibrosis stage in chronic hepatitis C. World J Gastroenterol.

[108] Robic MA, Procopet B, Métivier S, Péron JM, Selves J, Vinel JP (2011). Liver stiffness accurately predicts portal hypertension related complications in patients with chronic liver disease: a prospective study. J Hepatol.

[109] Lunova M, Frankova S, Gottfriedova H, Senkerikova R, Neroldova M, Kovac J (2021). Portal hypertension is the main driver of liver stiffness in advanced liver cirrhosis. Physiol Res.

[110] Kechagia JZ, Ivaska J, Roca-Cusachs P (2019). Integrins as biomechanical sensors of the microenvironment. Nat Rev Mol Cell Biol.

[111] Harburger DS, Calderwood DA (2009). Integrin signalling at a glance. J Cell Sci.

[112] Moretti L, Stalfort J, Barker TH, Abebayehu D (2022). The interplay of fibroblasts, the extracellular matrix, and inflammation in scar formation. J Biol Chem.

[113] Maldonado H, Hagood JS (2021). Cooperative signaling between integrins and growth factor receptors in fibrosis. J Mol Med.

[114] Bonnans C, Chou J, Werb Z (2014). Remodelling the extracellular matrix in development and disease. Nat Rev Mol Cell Biol.

[115] Onodera T, Sakai T, Hsu JC, Matsumoto K, Chiorini JA, Yamada KM (2010). Btbd7 regulates epithelial cell dynamics and branching morphogenesis. Science.

[116] Wegener KL, Partridge AW, Han J, Pickford AR, Liddington RC, Ginsberg MH (2007). Structural basis of integrin activation by talin. Cell.

[117] Kim CH, Ye F, Hu XH, Ginsberg MH (2012). Talin activates integrins by altering the topology of the β transmembrane domain. J Cell Biol.

[118] Mitra SK, Schlaepfer DD (2006). Integrin-regulated FAK-Src signaling in normal and cancer cells. Curr Opin Cell Biol.

[119] Clemente CFMZ, Tornatore TF, Theizen TH, Deckmann AC, Pereira TC, Lopes-Cendes I (2007). Targeting focal adhesion kinase with small interfering RNA prevents and reverses load-induced cardiac hypertrophy in mice. Circ Res.

[120] Dupont S (2016). Role of YAP/TAZ in cell-matrix adhesion-mediated signalling and mechanotransduction. Exp Cell Res.

[121] Dupont S, Morsut L, Aragona M, Enzo E, Giulitti S, Cordenonsi M (2011). Role of YAP/TAZ in mechanotransduction. Nature.

[122] Mohri Z, Hernandez AD, Krams R (2017). The emerging role of YAP/TAZ in mechanotransduction. J Thorac Dis.

[123] Ritsvall O, Albinsson S (2023). Emerging role of YAP/TAZ in vascular mechanotransduction and disease. Microcirculation.

[124] Lampi MC, Reinhart-King CA (2018). Targeting extracellular matrix stiffness to attenuate disease: from molecular mechanisms to clinical trials. Sci Transl Med..

[125] Liu F, Lagares D, Choi KM, Stopfer L, Marinkovic A, Vrbanac V (2015). Mechanosignaling through YAP and TAZ drives fibroblast activation and fibrosis. Am J Physiol-Lung Cell Mol Physiol.

[126] Du J, Zu Y, Li J, Du SY, Xu YP, Zhang L (2016). Extracellular matrix stiffness dictates Wnt expression through integrin pathway. Sci Rep.

[127] Bera K, Kiepas A, Godet I, Li YZ, Mehta P, Ifemembi B (2022). Extracellular fluid viscosity enhances cell migration and cancer dissemination. Nature.

[128] Xia HT, Zahra A, Jia M, Wang Q, Wang YF, Campbell SL (2022). Na+/H+ exchanger-1, a potential therapeutic drug target for cardiac hypertrophy and heart failure. Pharmaceuticals.

[129] Gonzalez-Molina J, Zhang XL, Borghesan M, da Silva JM, Awan M, Fuller B (2018). Extracellular fluid viscosity enhances liver cancer cell mechanosensing and migration. Biomaterials.

[130] Kim DH, Wong PK, Park J, Levchenko A, Sun Y (2009). Microengineered platforms for cell mechanobiology. Annu Rev Biomed Eng.

[131] Vernerey FJ, Sridhar SL, Muralidharan A, Bryant SJ (2021). Mechanics of 3D cell-hydrogel interactions: experiments, models, and mechanisms. Chem Rev.

[132] Rodriguez ML, McGarry PJ, Sniadecki NJ (2013). Review on cell mechanics: experimental and modeling approaches. Appl Mech Rev.

[133] Baker BM, Chen CS (2012). Deconstructing the third dimension - how 3D culture microenvironments alter cellular cues. J Cell Sci.

[134] Leung CM, de Haan P, Ronaldson-Bouchard K, Kim GA, Ko J, Rho HS (2022). A guide to the organ-on-a-chip. Nat Rev Method Prim.

[135] Low LA, Mummery C, Berridge BR, Austin CP, Tagle DA (2021). Organs-on-chips: into the next decade. Nat Rev Drug Discov.

[136] Bhatia SN, Ingber DE (2014). Microfluidic organs-on-chips. Nat Biotechnol.

[137] Zhang B, Radisic M (2017). Organ-on-a-chip devices advance to market. Lab Chip.

[138] Ekert JE, Deakyne J, Pribul-Allen P, Terry R, Schofield C, Jeong CG (2020). Recommended guidelines for developing, qualifying, and implementing complex in vitro models (CIVMs) for drug discovery. SLAS Discov.

[139] Kopec AK, Yokokawa R, Khan N, Horii I, Finley JE, Bono CP (2021). Microphysiological systems in early stage drug development: Perspectives on current applications and future impact. J Toxicol Sci.

[140] Nel AE, Mädler L, Velegol D, Xia T, Hoek EMV, Somasundaran P (2009). Understanding biophysicochemical interactions at the nano-bio interface. Nat Mater.

[141] Zhu MT, Nie GJ, Meng H, Xia T, Nel A, Zhao YL (2013). Physicochemical properties determine nanomaterial cellular uptake, transport, and fate. Accounts Chem Res.

[142] Villanueva-Flores F, Castro-Lugo A, Ramírez OT, Palomares LA (2020). Understanding cellular interactions with nanomaterials: towards a rational design of medical nanodevices. Nanotechnology.

[143] Foroozandeh P, Aziz AA (2018). Insight into cellular uptake and intracellular trafficking of nanoparticles. Nanoscale Res Lett.

[144] Byakodi M, Shrikrishna NS, Sharma R, Bhansali S, Mishra Y, Kaushik A (2022). Emerging 0D, 1D, 2D, and 3D nanostructures for efficient point-of-care biosensing. Biosens Bioelectron: X.

[145] Agarwal R, Singh V, Jurney P, Shi L, Sreenivasan SV, Roy K (2013). Mammalian cells preferentially internalize hydrogel nanodiscs over nanorods and use shape-specific uptake mechanisms. Proc Natl Acad Sci USA.

[146] Decuzzi P, Pasqualini R, Arap W, Ferrari M (2009). Intravascular delivery of particulate systems: does geometry really matter?. Pharm Res.

[147] Decuzzi P, Ferrari M (2006). The adhesive strength of non-spherical particles mediated by specific interactions. Biomaterials.

[148] Vácha R, Martinez-Veracoechea FJ, Frenkel D (2011). Receptor-mediated endocytosis of nanoparticles of various shapes. Nano Lett.

[149] Li Y, Kröger M, Liu WK (2015). Shape effect in cellular uptake of PEGylated nanoparticles: comparison between sphere, rod, cube and disk. Nanoscale.

[150] Zhang X, Ma GH, Wei W (2021). Simulation of nanoparticles interacting with a cell membrane: probing the structural basis and potential biomedical application. Npg Asia Materials.

[151] Yang K, Ma YQ (2010). Computer simulation of the translocation of nanoparticles with different shapes across a lipid bilayer. Nat Nanotechnol.

[152] Gupta R, Badhe Y, Mitragotri S, Rai B (2020). Permeation of nanoparticles across the intestinal lipid membrane: dependence on shape and surface chemistry studied through molecular simulations. Nanoscale.

[153] Shen ZQ, Ye HL, Yi X, Li Y (2019). Membrane wrapping efficiency of elastic nanoparticles during endocytosis: size and shape matter. ACS Nano.

[154] Li Y, Yue TT, Yang K, Zhang XR (2012). Molecular modeling of the relationship between nanoparticle shape anisotropy and endocytosis kinetics. Biomaterials.

[155] Anselmo AC, Zhang M, Kumar S, Vogus DR, Menegatti S, Helgeson ME (2015). Elasticity of nanoparticles influences their blood circulation, phagocytosis, endocytosis, and targeting. ACS Nano.

[156] Yi X, Shi XH, Gao HJ (2011). Cellular uptake of elastic nanoparticles. Phys Rev Lett.

[157] Shen ZQ, Ye HL, Li Y (2018). Understanding receptor-mediated endocytosis of elastic nanoparticles through coarse grained molecular dynamic simulation. Phys Chem Chem Phys.

[158] Tang HY, Ye HF, Zhang HW, Zheng YG (2015). Wrapping of nanoparticles by the cell membrane: the role of interactions between the nanoparticles. Soft Matter.

[159] Yi X, Gao HJ (2017). Kinetics of receptor-mediated endocytosis of elastic nanoparticles. Nanoscale.

[160] Banquy X, Suarez F, Argaw A, Rabanel JM, Grutter P, Bouchard JF (2009). Effect of mechanical properties of hydrogel nanoparticles on macrophage cell uptake. Soft Matter.

[161] Guo P, Liu DX, Subramanyam K, Wang BR, Yang J, Huang J (2018). Nanoparticle elasticity directs tumor uptake. Nat Commun.

[162] Sun JS, Zhang L, Wang JL, Feng Q, Liu DB, Yin QF (2015). Tunable rigidity of (polymeric core)-(lipid shell) nanoparticles for regulated cellular uptake. Adv Mater.

[163] Geraghty RJ, Capes-Davis A, Davis JM, Downward J, Freshney RI, Knezevic I (2014). Guidelines for the use of cell lines in biomedical research. Br J Cancer.

[164] Kaur G, Dufour JM (2012). Cell lines: valuable tools or useless artifacts. Cell lines Spermatogen.

[165] Richter M, Piwocka O, Musielak M, Piotrowski I, Suchorska WM, Trzeciak T (2021). From donor to the lab: a fascinating journey of primary cell lines. Front Cell Dev Biol.

[166] Hughes P, Marshall D, Reid Y, Parkes H, Gelber C (2007). The costs of using unauthenticated, over-passaged cell lines: how much more data do we need?. Biotechniques.

[167] Masters JRW (2010). Cell line misidentification: the beginning of the end. Nat Rev Cancer.

[168] Lorsch JR, Collins FS, Lippincott-Schwartz J (2014). Fixing problems with cell lines. Science.

[169] Souren NY, Fusenig NE, Heck S, Dirks WG, Capes-Davis A, Bianchini F (2022). Cell line authentication: a necessity for reproducible biomedical research. EMBO J.

[170] Fusenig NE, Capes-Davis A, Bianchini F, Sundell S, Lichter P (2017). The need for a worldwide consensus for cell line authentication: Experience implementing a mandatory requirement at the. PLoS Biol.

[171] Ben-David U, Siranosian B, Ha G, Tang H, Oren Y, Hinohara K (2018). Genetic and transcriptional evolution alters cancer cell line drug response. Nature.

[172] Liu YS, Mi Y, Mueller T, Kreibich S, Williams EG, Van Drogen A (2019). Multi-omic measurements of heterogeneity in HeLa cells across laboratories. Nat Biotechnol.

[173] Marx V (2014). Cell-line authentication demystified. Nat Methods.

[174] Pan CP, Kumar C, Bohl S, Klingmueller U, Mann M (2009). Comparative proteomic phenotyping of cell lines and primary cells to assess preservation of cell type-specific functions. Mol Cell Proteomics.

[175] Alge CS, Hauck SM, Priglinger SG, Kampik A, Ueffing M (2006). Differential protein profiling of primary versus immortalized human RPE cells identifies expression patterns associated with cytoskeletal remodeling and cell survival. J Proteome Res.

[176] Wei X, Wei R, Jiang GY, Jia YJ, Lou H, Yang ZY (2019). Mechanical cues modulate cellular uptake of nanoparticles in cancer via clathrin-mediated and caveolae-mediated endocytosis pathways. Nanomedicine.

[177] Wei Q, Huang CJ, Zhang Y, Zhao TK, Zhao P, Butler P (2018). Mechanotargeting: mechanics-dependent cellular uptake of nanoparticles. Adv Mater.

[178] Panzetta V, Guarnieri D, Paciello A, Della Sala F, Muscetti O, Raiola L (2017). ECM mechano-sensing regulates cytoskeleton assembly and receptor-mediated endocytosis of nanoparticles. ACS Biomater Sci Eng.

[179] Voigt JL, Timmer J, Pennarola F, Christian J, Meng N, Blumberg JW (2023). Substrate stiffness and particle properties influence cellular uptake of nanoparticles and viruses from the ventral side. Adv Funct Mater.

[180] Davis JR, Tapon N (2019). Hippo signalling during development. Development.

[181] Rausch V, Hansen CG (2020). The Hippo pathway, YAP/TAZ, and the plasma membrane. Trends Cell Biol.

[182] Strippoli R, Sandoval P, Moreno-Vicente R, Rossi L, Battistelli C, Terri M (2020). Caveolin1 and YAP drive mechanically induced mesothelial to mesenchymal transition and fibrosis. Cell Death Dis.

[183] Abostait A, Tyrrell J, Abdelkarim M, Shojaei S, Tse WH, El-Sherbiny IM (2022). Placental nanoparticle uptake-on-a-chip: the impact of trophoblast syncytialization and shear stress. Mol Pharm.

[184] Rinkenauer AC, Press AT, Raasch M, Pietsch C, Schweizer S, Schwörer S (2015). Comparison of the uptake of methacrylate-based nanoparticles in static and dynamic systems as well as. J Control Release.

[185] Jurney P, Agarwal R, Singh V, Choi D, Roy K, Sreenivasan SV (2017). Unique size and shape-dependent uptake behaviors of non-spherical nanoparticles by endothelial cells due to a shearing flow. J Control Release.

[186] Hosta-Rigau L, Städler B (2013). Shear stress and its effect on the interaction of myoblast cells with nanosized drug delivery vehicles. Mol Pharm.

[187] Chen YY, Syed AM, MacMillan P, Rocheleau JV, Chan WCW (2020). Flow rate affects nanoparticle uptake into endothelial cells. Adv Mater.

[188] Palchetti S, Pozzi D, Capriottic AL, La Barbera G, Chiozzi RZ, Digiacomo L (2017). Influence of dynamic flow environment on nanoparticle-protein corona: From protein patterns to uptake in cancer cells. Colloid Surf B-Biointerfaces.

[189] Samuel SP, Jain N, O'Dowd F, Paul T, Kashanin D, Gerard VA (2012). Multifactorial determinants that govern nanoparticle uptake by human endothelial cells under flow. Int J Nanomed.

[190] Fede C, Albertin G, Petrelli L, De Caro R, Fortunati I, Weber V (2017). Influence of shear stress and size on viability of endothelial cells exposed to gold nanoparticles. J Nanopart Res.

[191] Yazdimamaghani M, Barber ZB, Moghaddam SPH, Ghandehari H (2018). Influence of silica nanoparticle density and flow conditions on sedimentation, cell uptake, and cytotoxicity. Mol Pharm.

[192] Tsai CL, Huang CY, Lu YC, Pai LM, Horák D, Ma YH (2022). Cyclic strain mitigates nanoparticle internalization by vascular smooth muscle cells. Int J Nanomed.

[193] Rouse JG, Haslauer CM, Loboa EG, Monteiro-Riviere NA (2008). Cyclic tensile strain increases interactions between human epidermal keratinocytes and quantum dot nanoparticles. Toxicol Vitro.

[194] Torosean S, Flynn B, Axelsson J, Gunn J, Samkoe KS, Hasan T (2013). Nanoparticle uptake in tumors is mediated by the interplay of vascular and collagen density with interstitial pressure. Nanomed-Nanotechnol Biol Med.

[195] Zhang HP, Hu ZQ, Wang JX, Xu JX, Wang XX, Zang GC (2023). Shear stress regulation of nanoparticle uptake in vascular endothelial cells. Regen Biomater..

[196] Zhao YX, Ye ZY, Liu YL, Zhang JJ, Kuermanbayi S, Zhou Y (2024). Investigating the role of extracellular matrix stiffness in modulating the ferroptosis process in hepatocellular carcinoma cells via scanning electrochemical microscopy. Anal Chem.

[197] Mai Z, Lin Y, Lin P, Zhao X, Cui L (2024). Modulating extracellular matrix stiffness: a strategic approach to boost cancer immunotherapy. Cell Death Dis.

[198] Tilghman RW, Blais EM, Cowan CR, Sherman NE, Grigera PR, Jeffery ED (2012). Matrix rigidity regulates cancer cell growth by modulating cellular metabolism and protein synthesis. PLoS ONE.

[199] Yeh YC, Ling JY, Chen WC, Lin HH, Tang MJ (2017). Mechanotransduction of matrix stiffness in regulation of focal adhesion size and number: reciprocal regulation of caveolin-1 and β1 integrin. Sci Rep.

[200] Li X, Klausen LH, Zhang W, Jahed Z, Tsai CT, Li TL (2021). Nanoscale surface topography reduces focal adhesions and cell stiffness by enhancing integrin endocytosis. Nano Lett.

[201] Mason DE, Collins JM, Dawahare JH, Nguyen TD, Lin Y, Voytik-Harbin SL (2019). YAP and TAZ limit cytoskeletal and focal adhesion maturation to enable persistent cell motility. J Cell Biol.

[202] Qin X, Zhang K, Qiu JH, Wang N, Qu K, Cui YL (2022). Uptake of oxidative stress-mediated extracellular vesicles by vascular endothelial cells under low magnitude shear stress. Bioact Mater.

[203] Cheng MJ, Mitra R, Okorafor CC, Nersesyan AA, Harding IC, Bal NN (2020). Targeted intravenous nanoparticle delivery: role of flow and endothelial glycocalyx integrity. Ann Biomed Eng.

[204] Yamamoto K, Ando J (2013). Endothelial cell and model membranes respond to shear stress by rapidly decreasing the order of their lipid phases. J Cell Sci.

[205] Mészáros M, Porkoláb G, Kiss L, Pilbat AM, Kóta Z, Kupihár Z (2018). Niosomes decorated with dual ligands targeting brain endothelial transporters increase cargo penetration across the blood-brain barrier. Eur J Pharm Sci.

[206] Charwat V, Calvo IO, Rothbauer M, Kratz SRA, Jungreuthmayer C, Zanghellini J (2018). Combinatorial in vitro and in silico approach to describe shear-force dependent uptake of nanoparticles in microfluidic vascular models. Anal Chem.

[207] Xu YY, Qin S, Niu YN, Gong T, Zhang ZR, Fu Y (2020). Effect of fluid shear stress on the internalization of kidney-targeted delivery systems in renal tubular epithelial cells. Acta Pharm Sin B.

[208] Freese C, Schreiner D, Anspach L, Bantz C, Maskos M, Unger RE (2014). In vitro investigation of silica nanoparticle uptake into human endothelial cells under physiological cyclic stretch. Part Fibre Toxicol.

[209] Shurbaji S, Anlar GG, Hussein EA, Elzatahry A, Yalcin HC (2020). Effect of flow-induced shear stress in nanomaterial uptake by cells: Focus on targeted anti-cancer therapy. Cancers.

[210] Frtus A, Smolkov B, Uzhytchak M, Lunova M, Jirsa M, Petrenko Y (2023). Mechanical regulation of mitochondrial dynamics and function in a 3D-engineered liver tumor microenvironment. ACS Biomater Sci Eng.

[211] Edington CD, Chen WLK, Geishecker E, Kassis T, Soenksen LR, Bhushan BM (2018). Interconnected microphysiological systems for quantitative biology and pharmacology studies. Sci Rep.

[212] Bircsak KM, DeBiasio R, Miedel M, Alsebahi A, Reddinger R, Saleh A (2021). A 3D microfluidic liver model for high throughput compound toxicity screening in the OrganoPlate®. Toxicology.

[213] Tischenko K, Brill-Karniely Y, Steinberg E, Segev-Yekutiel H, Benny O (2023). Surface physical cues mediate the uptake of foreign particles by cancer cells. APL Bioeng.

[214] Hu J, Liu YL (2015). Cyclic strain enhances cellular uptake of nanoparticles. J Nanomater.

